# Comparison Study of Computational Prediction Tools for Drug-Target Binding Affinities

**DOI:** 10.3389/fchem.2019.00782

**Published:** 2019-11-20

**Authors:** Maha Thafar, Arwa Bin Raies, Somayah Albaradei, Magbubah Essack, Vladimir B. Bajic

**Affiliations:** ^1^Computer, Electrical and Mathematical Science and Engineering (CEMSE) Division, Computational Bioscience Research Center (CBRC), King Abdullah University of Science and Technology (KAUST), Thuwal, Saudi Arabia; ^2^College of Computers and Information Technology, Taif University, Taif, Saudi Arabia; ^3^Faculty of Computing and Information Technology, King Abdulaziz University, Jeddah, Saudi Arabia

**Keywords:** drug repurposing, drug-target interaction, drug-target binding affinity, artificial intelligence, machine learning, deep learning, information integration, bioinformatics

## Abstract

The drug development is generally arduous, costly, and success rates are low. Thus, the identification of drug-target interactions (DTIs) has become a crucial step in early stages of drug discovery. Consequently, developing computational approaches capable of identifying potential DTIs with minimum error rate are increasingly being pursued. These computational approaches aim to narrow down the search space for novel DTIs and shed light on drug functioning context. Most methods developed to date use binary classification to predict if the interaction between a drug and its target exists or not. However, it is more informative but also more challenging to predict the strength of the binding between a drug and its target. If that strength is not sufficiently strong, such DTI may not be useful. Therefore, the methods developed to predict drug-target binding affinities (DTBA) are of great value. In this study, we provide a comprehensive overview of the existing methods that predict DTBA. We focus on the methods developed using artificial intelligence (AI), machine learning (ML), and deep learning (DL) approaches, as well as related benchmark datasets and databases. Furthermore, guidance and recommendations are provided that cover the gaps and directions of the upcoming work in this research area. To the best of our knowledge, this is the first comprehensive comparison analysis of tools focused on DTBA with reference to AI/ML/DL.

## Introduction

Experimental confirmation of new drug-target interactions (DTIs) is not an easy task, as *in vitro* experiments are laborious and time-consuming. Even if a confirmed DTI has been used for developing a new drug (in this review compounds that are not approved drugs are also referred to as drugs), the approval for human use of such new drugs can take many years and estimated cost may run over a billion US dollars (Dimasi et al., 2003). Moreover, although huge investments are required for the development of novel drugs, they are often met with failure. In fact, of the 108 new and repurposed drugs reported as Phase II failures between 2008 and 2010, 51% was due to insufficient efficacy as per a Thomson Reuters Life Science Consulting report (Arrowsmith, 2011). This observation highlighted the need for: (1) new, more appropriate drug targets, and (2) *in silico* methods that can improve the efficiency of the drug discovery and screen a large number of drugs in the very initial phase of drug discovery process, thus guiding toward those drugs that may exhibit better efficacy. In this regard, methods that predict DTIs and specifically, drug-target binding affinities (DTBA) are of great interest.

Over the last three decades, several methods that predict DTIs have been developed ranging from ligand/receptor-based methods (Cheng et al., 2007; Wang et al., 2013) to gene ontology-based (Mutowo et al., 2016), text-mining-based methods (Zhu et al., 2005), and reverse virtual screening techniques (reverse-docking) (Lee et al., 2016; Vallone et al., 2018; Wang et al., 2019). Development of such methods is still ongoing as each method suffers from different types of limitations. For example, docking simulation is often used in the receptor-based methods; also, docking simulation requires the 3D structures of the target proteins that are not always readily available. Furthermore, this is an expensive process. On the other hand, the ligand-based approaches suffer from low performance when the number of known ligands of target proteins is small, as this approach predicts DTIs based on the similarity between candidate ligands and the known ligands of the target proteins. The limitations associated with gene ontology-based and text-mining-based approaches are the same, their major limitation appears to be what is reported in the text. This also becomes more complicated due to the frequent use of redundant names for drugs and target proteins. Moreover, with the text-mining approach being limited to the current knowledge (i.e., published material), making discovery of new knowledge is not easy.

Other methods such as deep learning (DL), machine learning (ML), and artificial intelligence (AI) in general, avoid these limitations by using models that learn the features of known drugs and their targets to predict new DTIs. Understanding that ML methods are just a subset of AI methods, does not always makes it clear what would be strictly an ML method and what an AI method. This particularly becomes apparent when graph, network, and search analyses methods are combined with conventional (shallow) ML approaches. The situation for DL is clearer, as these methods are a subset of ML approaches based on transformation of the original input data representation across multiple information processing layers, thus distinguishing them from the shallow ML approaches. More recent approaches introduced AI, network analysis, and graph mining (Emig et al., 2013; Ba-Alawi et al., 2016; Luo et al., 2017; Olayan et al., 2018), and ML and DL techniques (Liu Y. et al., 2016; Rayhan et al., 2017; Zong et al., 2017; Tsubaki et al., 2019) to develop prediction models for DTI problem. AI/ML-based methods (we will frequently refer to them in this study as ML methods) are generally feature-based or similarity-based (see DTBA ML-based methods section). Feature-based AI/ML methods can be integrated with other approaches constructing “Ensemble system” as presented in Ezzat et al. (2016), Jiang et al. (2017), and Rayhan et al. (2019). Thus, several comprehensive recent reviews summarized the different studies that predict DTIs using various techniques covering structure-based, similarity-based, network-based, and AI/ML-based methods as presented in Liu Y. et al. (2016), Ezzat et al. (2017, 2018, 2019), Rayhan et al. (2017), Trosset and Cavé (2019), and Wan et al. (2019). Other reviews focused on one aspect which are similarity-based methods (Ding et al., 2014; Kurgan and Wang, 2018) or feature-based methods (Gupta, 2017). Most of the approaches mentioned above address DTI prediction as a simple binary on-off relationship. That is, they simply predict whether the drug and target could interact or not. This approach suffers from two major limitations including: (1) the inability to differentiate between true negative interactions and instances where the lack of information or missing values impede predicting an interaction, and (2) it does not reflect how tightly the drug binds to the target which reflects the potential efficacy of the drug. To overcome these limitations, approaches that focus on DTBA predictions have been developed. We compile this study with the focus on DTBA, which has not been addressed well in the past, but is more critical for estimating usefulness of DTI in early stages of drug development.

## Drug-Target Binding Affinity (DTBA)

DTBA indicates the strength of the interaction or binding between a drug and its target (Ma W. et al., 2018). The advantage of formulating drug-target prediction as a binding affinity regression task, is that it can be transformed from regression to either binary classification by setting specific thresholds or to ranking problem (He et al., 2017). This enables different generalization options.

**Figure 1:**
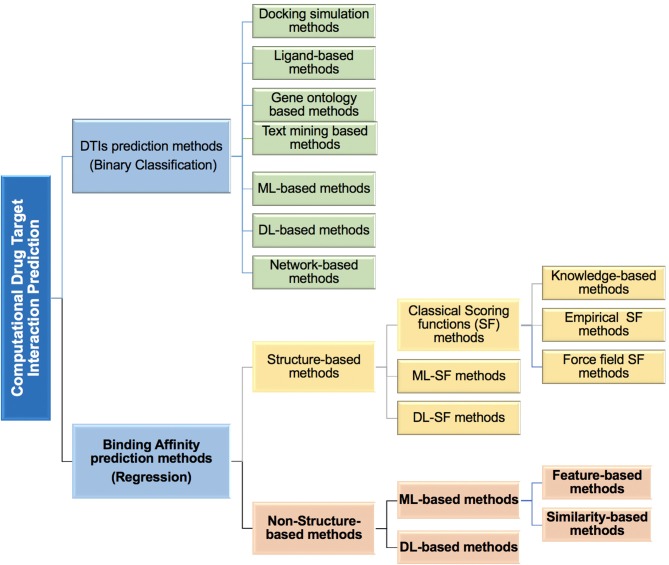
An overview of the different types of computational methods developed to predict drug-target interactions (DTIs) and drug-target binding affinity (DTBA) categories.

Most *in silico* DTBA prediction methods developed to date use 3D structural information (see Figure 1), which was demonstrated to successfully contribute to the drug design (Leach et al., 2006). Some of these methods provide free analysis software as reported by Agrawal et al. (2018). The 3D structure information of proteins is used in the molecular docking analysis and followed by applying search algorithms or scoring functions to assist with the binding affinity predictions (Scarpino et al., 2018; Sledz and Caflisch, 2018). This whole process is used in the structured-based virtual screening (Li and Shah, 2017).

In DTBA predictions, the concept of scoring function (SF) is frequently used. SF reflects the strength of binding affinity between ligand and protein interaction (Abel et al., 2018). When SFs have a prearranged functional form that mimics the relationship between structural features and binding affinity, it is called classical SF. Classical SFs are categorized as Empirical SFs (Guedes et al., 2018), Force field SFs (Huang and Zou, 2006), and Knowledge-based SFs (Huang and Zou, 2006; Liu et al., 2013). SFs have been used to predict protein-ligand interaction in molecular docking such as with the Binding Estimation After Refinement (BEAR) SF (Degliesposti et al., 2011) which is a post docking tool that uses molecular dynamics to accurately predict protein binding free energies using SF. Several of these classical SFs are summarized in a recent review (Li J. et al., 2019). A specific form of the SF called target-specific SF, is based on energy calculations of interacting compound (i.e., free energy calculations; Ganotra and Wade, 2018; Sun et al., 2018). Other SFs were also developed that do not follow a predetermined functional form. These SFs use ML techniques to infer functional form from training data (Deng et al., 2004; Vert and Jacob, 2008; Kundu et al., 2018). Thus, the ML-based SFs methods are data-driven models that capture the non-linearity relationship in data making the SF more general and more accurate. DL is an emerging research area in different cheminformatic fields including drug design (Jain, 2017; Andricopulo and Ferreira, 2019). SFs that use DL in structure-based methods focused on binding affinity prediction have been developed (Ashtawy and Mahapatra, 2018; Jiménez et al., 2018; Antunes et al., 2019). As all DL models, these DL-based SFs methods learn the features to predict binding affinity without requirement for feature engineering as may be the case in the ML methods. Several reviews have been made covering virtual screening structure-based binding affinity prediction methods including docking techniques, before applying SFs (Kontoyianni, 2017; Li and Shah, 2017), classical SFs (Guedes et al., 2018), or ML-derived SFs (Ain et al., 2015; Heck et al., 2017; Colwell, 2018; Kundu et al., 2018). The main limitations of the structure-based methods are the requirement for the 3D structure data (including compound and protein) that are scarce. This is compounded by the problem of low-quality structure predicted from docking, which cannot be tested and scaled to large-scale data applications (Karimi et al., 2019). Several publications have discussed the major limitations of structured-based virtual screening (Sotriffer and Matter, 2011; Hutter, 2018).

Non-structure-based methods, overcome most of these limitations since there is no need for the docking process or 3D structural data. Despite the enormous amount of effort and research devoted to binding affinity prediction, there are only a few publications that address the DTBA problem as a non-structure-based approach. This remains a critical and challenging task that requires the development of significantly improved algorithms.

Here, we review methods developed for prediction of DTIs based on binding affinities. Specifically, we focus on the novel methods that utilize non-structure-based binding affinity prediction (shown in bold font in Figure 1), which does not require or use 3D structural data. The study provides a comparative analysis of the current DTBA prediction methods. It covers: (a) definitions and calculations associated with binding affinity, (b) the benchmark datasets that are used in DTBA regression problem, (c) computational methods used, (d) evaluation and performance comparison of DTBA prediction methods, and (e) recommendations of areas for improvement and directions in binding affinity prediction research.

## Measuring Binding Affinity

Each ligand/protein has a unique binding affinity constant for specific receptor system which can be used to identify distinct receptors (Weiland and Molinoff, 1981; Bulusu et al., 2016). The equilibrium reaction below describes how a protein (*P*) binds to its ligand (*L*) to create the protein-ligand complex (*PL*) (Du et al., 2016):

(1) $$\begin{array}{l} P+L\overset{k_{\alpha }}{\Leftrightarrow }PL \end{array}$$

*K*_*a*_ is the equilibrium association constant (also called binding affinity constant). A high value of *K*_*a*_ indicates a strong binding capacity between the drug/ligand and the receptor/protein (Weiland and Molinoff, 1981; Bulusu et al., 2016). The inverse of the above reaction is when the protein-ligand complex dissociates into its components of a protein and a ligand as explained in the equilibrium reaction below (Du et al., 2016):

(2) $$\begin{array}{l} PL\overset{K_{d}}{\Leftrightarrow }P+L \end{array}$$

*K*_*d*_ is the equilibrium dissociation constant, and it is used more often than *K*_*a*_. Small values of *K*_*d*_ indicate higher affinity (Ma W. et al., 2018). *K*_*d*_ is the inverse of the *K*_*a*_ as illustrated in the equation below (Du et al., 2016):

(3) $$\begin{array}{l} K_{d}=\frac{1}{K_{a}} \end{array}$$

### Binding Curve

**Figure 2:**
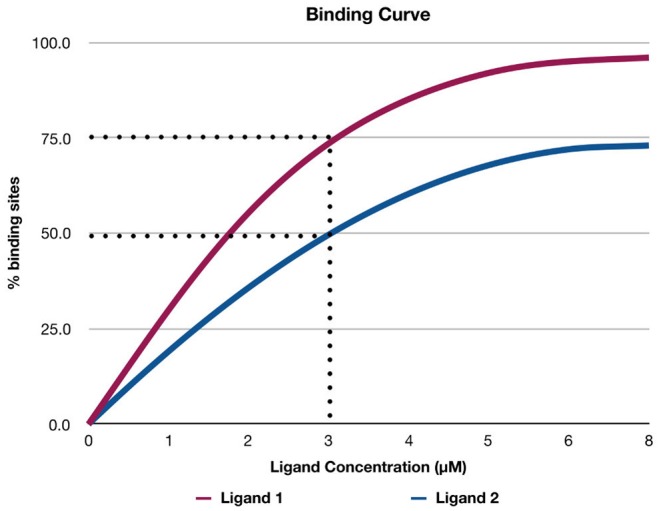
A hypothetical example of a binding curve for ligand 1 and ligand 2. The *x*-axis shows the concentration of the ligand, and the *y*-axis shows the percentage of available binding sites (Θ) in a protein that is occupied by the ligand.

Figure 2 shows a hypothetical example of a binding curve for two ligands: Ligand 1 and Ligand 2. The *x*-axis represents the concentration of the ligand, and the *y*-axis represents the percentage of available binding sites (Θ) in a protein that is occupied by the ligand. The values of Θ range from 0 to 1 (corresponding to the range from 0 to 100% in Figure 2). For example, if Θ is 0.5, this means that 50% of the available binding sites are occupied by the ligand. The binding curves help in determining graphically which ligand binds more strongly to the protein at a specific concentration of the ligand (Stefan and Le Novère, 2013). For example, in Figure 2, if the concentration of the ligands is 3 μ*M*, Ligand 1 binds to 75% of the binding sites of the protein, while Ligand 2 binds to only 50% of the binding sites. Therefore, Ligand 1 binds more strongly to the protein than Ligand 2. Figure 2 depicts an example of cooperative binding (if the concentration of the ligand increases, the number of binding sites the ligand occupies increases non-linearly). Cooperative binding is positive if binding of the ligand increases the affinity of the protein and increases the chance of another ligand binding to the protein; otherwise, the cooperative binding is negative (i.e., binding of the ligand to the protein decreases the affinity of the protein and reduces the chance of another ligand binding to the protein; Stefan and Le Novère, 2013).

The equation below shows the relationship between Θ for a protein to which the ligand binds, and *K*_*d*_ of the equilibrium reaction at a given concentration of the ligand [*L*] (Salahudeen and Nishtala, 2017):

(4) $$\begin{array}{l} \theta =\frac{\left[L\right]}{K_{d}+\left[L\right]} \end{array}$$

### *K*_*i*_ and IC_50_ Constants

**Figure 3:**
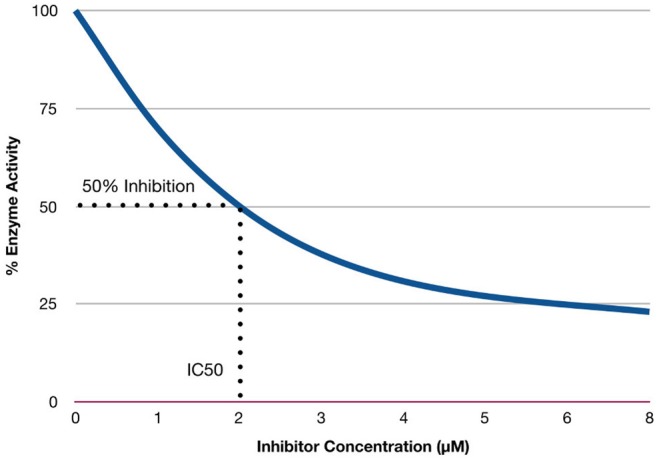
Relationship between concentration of inhibitors and enzymes activity.

The inhibitor constant (*K*_*i*_) is an indicator of the potency of an inhibitor (Bachmann and Lewis, 2005). Inhibitors are compounds (e.g., drugs) that can reduce the activity of enzymes. Enzymes that exhibit overactivity are potential targets for drugs to treat specific diseases, as well as inhibitors of a cascade of events in a pathway. Several drugs act by inhibiting these specific enzymes (Chou and Talalay, 1984; Tang et al., 2017). IC_50_ is the concentration required to produce half-maximum inhibition (Bachmann and Lewis, 2005). *K*_*i*_ is calculated using IC_50_ values, which are the concentration required to produce 50% inhibition (Burlingham and Widlanski, 2003). Figure 3 provides a hypothetical example of IC_50_ values, with the concentration of the inhibitor represented on the *x*-axis, and the percentage of enzyme activity represented on the *y*-axis. The hypothetical example (in Figure 3) shows 50% of enzyme activity can be inhibited when the concentration of the inhibitor is 2 μ*M*.

IC_50_ is not an indicator of affinity, but rather indicates the functional strength of the inhibitor. On the other hand, *K*_*i*_ constant reflects the binding affinity of the inhibitor. Lower values of *K*_*i*_ indicate higher affinity. The relationship between IC_50_ and *K*_*i*_ is explained by the equation below (Hulme and Trevethick, 2010):

(5) $$\begin{array}{l} K_{i}=\frac{{IC}_{50}}{1+\;\frac{\left[S\right]}{K_{m}}} \end{array}$$

where *K*_*m*_ is the substrate concentration (in the absence of inhibitor) at which the velocity of the reaction is half-maximal, and [*S*] is the concentration of substrate. More details about *K*_*m*_ can be found in Hulme and Trevethick (2010).

## Benchmark Datasets and Sources

Benchmark datasets are used to train models and evaluate their performance on the standardized data. Using these datasets also allow the performance of the newly developed method to be compared to the state-of-the-art methods to establish the best performance. Only a few benchmark datasets have been used to develop *in silico* DTBA prediction methods. When predicting DTIs, the Yamanishi datasets (Yamanishi et al., 2008) are the most popular benchmark datasets. There are four Yamanishi datasets based on family of target proteins, including: (1) nuclear receptors (NR), (2) G protein-coupled receptors (GPCR), (3) ion channels (IC), and (4) enzymes (E). Each dataset contains binary labels to indicate the interacting or non-interacting drug-target pairs (Yamanishi et al., 2008). However, these datasets cannot be used for DTI regression-based models, because the datasets do not indicate the actual binding affinities between known interacting drug-target pairs. That is, actual binding affinity scores are needed to train the models to predict the continuous values that indicate the binding strength between drugs and their targets. Three large-scale benchmark datasets that we name Davis dataset, Metz dataset, and Kinase Inhibitor BioActivity (KIBA) dataset, which provide these binding affinities for interaction strength were used to evaluate DTBA prediction in Davis et al. (2011), Metz et al. (2011), and Tang et al. (2014), respectively. All three datasets are large scale biochemical selectivity assays of the kinase inhibitors. The kinase protein family is used for the reason that this protein family has increased biological activity and is involved in mediating critical pathway signals in cancer cells (Tatar and Taskin Tok, 2019).

In Davis dataset, the *K*_*d*_ value is provided as a measure of binding affinity. The Metz dataset provides the *K*_*i*_ as a measure of binding affinity. When the value of *K*_*d*_ or *K*_*i*_ is small, this indicates strong binding affinity between a drug and its target. KIBA dataset integrates different bioactivities and combines *K*_*d*_, *K*_*i*_, and IC_50_ measurements. KIBA score represents a continuous value of the binding affinity that was calculated utilizing *K*_*d*_, *K*_*i*_, and IC_50_ scores. The higher KIBA score indicates a lower binding affinity between a drug and its target.

Recently, Feng (2019) also used ToxCast (Judson, 2012) as a benchmark dataset for binding affinity. This dataset is much larger than the other three benchmark datasets. It contains data about different proteins that can help in evaluating the model robustness and scalability. ToxCast contains toxicology data obtained from *in vitro* high-throughput screening of drugs (i.e., chemicals). Several companies have done ToxCast curation with 61 different measurements of binding affinity scores. Other details of this dataset and the method are explained later in section Computational Prediction of Drug-Target Binding Affinities. Table 1 summarizes the statistics for these four benchmark datasets.

**Table 1:** Binding affinity benchmark datasets statistics.

**Datasets**	**No. of drugs**	**No. of proteins**	**Known DTIs**
Davis	68	442	30,056
Metz	1,421	156	35,259
Kiba	2,116	229	118,254
ToxCast	7,675	335	530,605

Other benchmark binding affinity datasets provided 3D structure information used to evaluate and validate structure-based methods via scoring functions and docking techniques. These benchmark datasets provide all the binding affinity information for the interactions. We listed these datasets without mentioning any further details since their use is beyond the scope of this study. Most of these datasets have more than one version since they are updated each year by adding more experimental, validated data. These datasets/data sources are: PDBbind (Wang et al., 2004, 2005), BindingDB (Chen et al., 2001; Liu et al., 2007; Gilson et al., 2016), BindingMOAD—(the Mother Of All Databases; Hu et al., 2005; Benson et al., 2007; Ahmed et al., 2015; Smith et al., 2019), CSAR (Smith et al., 2011; Dunbar et al., 2013), AffinDB (Block et al., 2006), Ligand Protein DataBase (LPDB) (Roche et al., 2001), and Protein-Ligand Database (PLD) (Puvanendrampillai and Mitchell, 2003). These datasets are integrated with protein 3D structure information provided in Protein Data Bank (PDB) (Berman et al., 2000; Westbrook et al., 2003) adding more information. All these resources are publicly available, and some of them have associated web-tools aiming to facilitate accessing and searching information.

## Computational Prediction of Drug-Target Binding Affinities

There are few cheminformatics methods developed to predict continuous DTBA that do not use the 3D structure data. These methods are data-driven and use AI/ML/DL techniques for regression task rather than classification. To our knowledge, there are only six state-of-art methods developed for DTBA prediction. These we describe in what follows.

### Artificial Intelligence and Machine Learning-Based Methods

AI/ML and statistical analysis approaches have been applied across different stages of the drug development and design pipelines (Lima et al., 2016) including target discovery (Ferrero et al., 2017), drug discovery (Hutter, 2009; Raschka et al., 2018; Vamathevan et al., 2019), multi-target drug combination prediction (Tang et al., 2014; Vakil and Trappe, 2019), and drug safety assessment (Raies and Bajic, 2016, 2018; Lu et al., 2018). AI/ML approaches are generally either feature-based or similarity-based. The feature-based approaches use known DTIs chemical descriptors for drugs and the descriptors for the targets to generate feature vectors. On the other hand, similarity-based AI/ML approaches use the “guilt by association” rule. Using this rule is based on the assumptions that similar drugs tend to interact with similar targets and similar targets are targeted by similar drugs. Such AI/ML approaches that predict binding affinity of DTIs were used to develop state-of-the-art DTBA prediction methods, KronRLS (Pahikkala et al., 2015) and SimBoost (He et al., 2017).

#### KronRLS

Regularized least-square (RLS) is an efficient model used in different types of applications (Pahikkala et al., 2012a,b). Van Laarhoven et al. (2011) used RLS for the binary prediction of DTIs and achieved outstanding performance. Later, the RLS model was amended to develop a method that is suitable for DTBA prediction named, Kronecker-Regularized Least Squares (KronRLS) (Pahikkala et al., 2015). This method is a similarity-based method that used different types of drug-drug similarity and protein-protein similarity score matrices as features. The problem is formulated as regression or rank prediction problem as follows: a set *D* of drugs {*d*_1_*, d*_2_*,., d*_*i*_} and a set *T* of protein targets {*t*_1_*, t*_2_*,., t*_*i*_} are given with the training data *X* = {*x*_1_*, x*_2_*,., x*_*n*_} that is a subset from all possible generated drug-target pairs *X* ⊂ {*d*_*i*_ × *t*_*j*_}. Each row of *X* (i.e., feature vector) is associated with the label *y*_*i*_, *y*_*i*_ ϵ *Y*_*n*_, where *Y*_*n*_ is the label vector that represents a binding affinity. To learn the prediction function *f*, a minimizer of the following objective function *J* is defined as:

(6) $$\begin{array}{l} J(f)=\sum_{i=1}^{m}(\;y_{i}\;-\;f(x_{i}){)}^{2}\;+\;\lambda {∥f∥}_{k}^{2} \end{array}$$

Here ||*f*||_*k*_ is the norm of *f*, λ > 0 is regularization parameter defined by the user, and *K* is the kernel function (i.e., similarity) that is associated with the norm. The objective function to be minimized during optimization process is defined as:

(7) $$\begin{array}{l} f\left(x\right)=\overset{m}{\underset{i=1}{\sum }}a_{i}K\left(x,x_{i}\right) \end{array}$$

The kernel function *K* in the equation above is the symmetric similarity matrix *n* × *n* for all possible drug-target pairs. This kernel function is the Kronecker product of two other similarity matrices: *K* = *K*_*d*_ ⊗ *K*_*t*_, where *K*_*d*_ is the drug chemical structure similarity matrix computed using the PubChem structure clustering tool, and *K*_*t*_ is the protein sequence similarity matrix computed using both original and normalized versions of the Smith-Waterman (SW) algorithm (Yamanishi et al., 2008; Ding et al., 2014). There are two scenarios of the training data. If the training set *X* = {*d*_*i*_ × *t*_*j*_} contains all possible pairs, the parameter vector *a* in Equation (7) can be obtained by solving the system of linear equations:

(8) $$\begin{array}{l} \left(K\;+\;I\right)\;a=y \end{array}$$

where *I* is the identity matrix. For the second scenario, if only a subset of {*d*_*i*_ × *t*_*j*_} is used as the training data, such as *X* ⊂ {*d*_*i*_ × *t*_*j*_}, the vector *y* has missing values for binding affinity and for determining the parameter *a*, conjugate gradient with Kronecker algebraic optimization is needed to solve the system of linear Equation (8).

#### SimBoost

SimBoost (He et al., 2017) is a novel non-linear method that has been developed to predict DTBA as a regression task using gradient boosting regression trees. This method uses both similarity matrices and constructed features. The definition of the training data is similar to the KronRLS method. Thus, SimBoost requires a set of, (1) drugs (D), (2) targets (T), (3) drug-target pairs (that are associated with user-defined features), and (4) binding affinity such that *y*_*i*_ ϵ *Y*_*n*_ (where *Y*_*n*_ is the binding affinity vector). SimBoost is used to generate features for each drug, target, and drug-target pair. There are three types of features:

Type-1 features are object-based features for every single drug and target. This type of features reflects the statistics and similarity information such as score, histogram, a frequency for every single object (drug or target).

Type-2 features are similar to network-based features. Here, two networks are built, one network for drug-drug similarity, and the other network for target-target similarity. For the drug-drug similarity network, each drug is a graph node, and the nodes connected through edges. Edges are determined using the similarity score that is higher than the user-defined threshold. The construction of the second target-target similarity network is similar to the drug-drug network. For each network, we extract features. These features include statistics of node neighbors, page rank, betweenness, and eigencentrality (introduced in Newman, 2018).

Type-3 features are heterogeneous network-based features from the drug-target network, where drugs and targets are connected based on binding affinity continuous value. We extract other features from this network such as the latent vectors using matrix factorization (Liu Y. et al., 2016), and the normal ones, including betweenness, closeness, and eigencentrality.

A feature vector is constructed for each (drug, target) pair by concatenating type-1 and type-2 feature vector for each *d*_*i*_ and *t*_*j*_ and type-3 feature vector for each pair (*d*_*i*_*, t*_*j*_). After finishing feature engineering, the feature vector is feed to the gradient boosting regression trees. In this model, the predicted score ŷ_*i*_ for each input data *x*_*i*_ that is represented by its feature vector, is computed using the following:

(9) $$\begin{array}{l} {ŷ}_{i}=\phi \left(x_{i}\right)=\overset{K}{\underset{k=1}{\sum }}f_{k}\left(x_{i}\right),f_{k}\;\in \;F \end{array}$$

Here, *B* is the number of regression trees, {*f*_*k*_} is the set of trees, and *F* represents the space of all possible trees. The following is the regularized objective function *L* used to learn the *f*_*k*_:

(10) $$\begin{array}{l} L\left(\phi \right)=\underset{i}{\sum }l\left({\hat{y}}_{i},\;y_{i}\right)\;+\underset{k}{\sum }\Omega \left(f_{k}\;\right) \end{array}$$

Here, *l* is a differentiable loss function that evaluates the prediction error. The Ω function measures the model complexity to avoid overfitting. The model is trained additively, at each iteration *t, F* is searched to find a new tree *f*_*t*_. This new tree *f*_*t*_ optimizes the following objective function:

(11) $$\begin{array}{r} L^{\left(t\right)}=\sum_{i\;=1}^{n}l(y_{i},\;{\hat{y}}_{i}^{\left(t\right)})\;+\sum_{i\;=1}^{t}\Omega (f_{i})=\sum_{i\;=1}^{t}l(y_{i},\;{\hat{y}}_{i}^{\left(t-1\right)}+\;f_{t}(x_{i}))\\ +\;\sum_{i\;=1}^{t}\Omega (f_{i}) \end{array}$$

A gradient boosting algorithm iteratively adds trees that optimize the approximate objective at specific step for several user-defined iterations. SimBoost used similarity matrices are the same as KronRLS and are obtained using drug-drug similarity (generated by PubChem clustering based on the chemical structure) and target-target similarity (generated using the SW algorithm based on protein sequences).

### Deep Learning-Based Methods

Recently and in this big data era, DL approaches have been successfully used to address diverse problems in bioinformatics/cheminformatics applications (Ekins, 2016; Kalkatawi et al., 2019; Li Y. et al., 2019) and more specifically in drug discovery as discussed in detail in Chen et al. (2018), Jing et al. (2018), and Ekins et al. (2019). DL algorithms developed to predict DTBA sometimes show superior performance when compared to conventional ML algorithms (Öztürk et al., 2018, 2019; Karimi et al., 2019). These DL-based algorithms developed to predict DTBA differ from each other in two main aspects. The first is concerning the representation of input data. For example, Simplified Molecular Input Line Entry System (SMILES), Ligand Maximum Common Substructure (LMCS) Extended Connectivity Fingerprint (ECFP), or a combination of these features can be used as drug features (see **Table 4**). The second is concerning the DL system architecture that is developed based on different neural network (NN) types (Krig, 2016) elaborated on below. The NN types differ in their structure that in some cases include the number of layers, hidden units, filter sizes, or the incorporated activation function. Each type of NN has its inherent unique strengths that make them more suitable for specific kinds of applications. The most popular NN types include the Feedforward Neural Network (FNN), Radial Basis Function Neural Network (RBNN), Multilayer Perceptron (MLP), Recurrent Neural Network (RNN), Convolutional Neural Network (CNN), and Modular Neural Network (MNN) (Schmidhuber, 2015; Liu et al., 2017). FNN and CNN have been used in algorithms discussed below to predict DTBA.

FNN, also known as a front propagated wave, is the simplest type of artificial NN (ANN) (Michelucci, 2018). In this type, the information only moves in one direction, from the input nodes to the output nodes, unlike more complex kinds of NN that have backpropagation. Nonetheless, it is not restricted to having a single layer, as it may have multiple hidden layers. Like all NN, FNN also incorporates an activation function. Activation function (Wu, 2009) is represented by a node which is added to the output layer or between two layers of any NN. Activation function node decides what output a neuron should produce, e.g., should it be activated or not. The form of the activation function is the non-linear transformation of the input signal to an output signal that serves as the input of a subsequent layer or the final output. Example of activation functions includes sigmoid, tanh, Rectified Linear Unit (ReLU), and variants of them.

On the other hand, CNN uses a variation of multilayer perceptron. Its architecture incorporates convolution layers which apply *k* filters on the input to systematically capture the presence of some discriminative features and create feature maps (Liu et al., 2017). Those filters are automatically learned based on the desired output, which maximizes the algorithms ability to identify true positive cases. This is achieved through a loss layer (loss function) which penalizes predictions based on their deviations from the training set. When many convolutional layers are stacked, more abstracted features are automatically detected. Usually, a pooling layer follows a convolution layer to limit the dimension and keep only the essential elements. The common types of pooling are average pooling and max pooling. Average pooling finds the average value for each patch on the feature map. Max pooling finds the maximum value for each patch of the feature map. The pooling layer produces a down-sampled feature map which reduces the computational cost. After features extraction and features selection automatically performed by the convolutional layers and pooling layers, fully connected layers are usually used to perform the final prediction.

**Figure 4:**
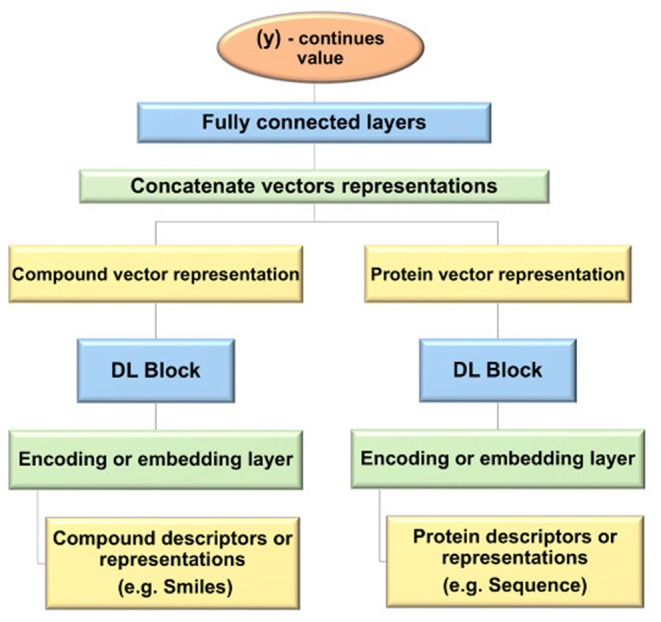
Flowchart of the general framework of deep learning (DL) models used for drug-target binding affinity (DTBA) prediction.

The general design used for the prediction of DTBA start with the representation of the input data for the drug and target, then different NN types with various structures are applied to learn features (i.e., embedding). Subsequently, the features of each drug-target pair are concatenated to create feature vectors for all drug-target pairs. The fully connected (FC) layers are fed with these feature vectors for the prediction task. Figure 4 provides a step-by-step depiction of this general framework.

#### DeepDTA

DeepDTA, introduced in Öztürk et al. (2018), is the first DL approach developed to predict DTBA, and it does not incorporate 3D structural data for prediction, i.e., it is non-structure-based method. DeepDTA uses SMILES, the one-dimensional representation of the drug chemical structure (Weininger, 1988, 1990), as representation of the drug input data for drugs, while the amino acid sequences are used to represent the input data for proteins. Integer/label encoding was used to encode drug SMILES. For example, the [1 3 63 1 63 5] label encodes the “CN = C = O” SMILES. The protein sequences are similarly encoded. More details about data preprocessing and representation are explained in Öztürk et al. (2018). A CNN (Liu et al., 2017) that contains three 1D convolutional layers following by max-pooling function (called the first CNN block) was applied on the drug embedding to learn latent features for each drug. All three 1D convolution layers in each CNN block consists of 32, 64, and 96 filters, respectively. An identical CNN block was constructed and applied on protein embedding as well. Subsequently, the feature vectors for each drug-target pair are concatenated and fed into the three FC layers coined DeepDTA. First two FC layers contain a similar number of hidden nodes equal to 1,024, and a dropout layer follows each one of them to avoid overfitting as a regularization technique, as introduced in Srivastava et al. (2014). The last FC layer has a smaller number of nodes equal to 512 that is followed by the output layer. ReLU (Nair and Hinton, 2010) layer is implements *J*(*x*) = *max*(*0, x*) that was used as the activation function (explained above). This model is following the general architecture that is illustrated in Figure 2, but with a different structure. Also, DeepDTA tunes several hyper-parameters such as the number of filters, filter length of the drug, filter length of the protein, hidden neurons number, batch size, dropout, optimizer, and learning rate in the validation step. The goal of this model is to minimize the difference between the predicted binding affinity value and the real binding affinity value in the training session. The goal of this model is to minimize the difference between the predicted binding affinity value and the real binding affinity value in the training session. DeepDTA performance significantly increased when using two CNN-blocks to learn feature representations of drugs and proteins. This study showed that performance is lower when using CNN to learn protein representation from the amino-acid sequence compared to other studies that are using CNN in their algorithms. This poor performance suggests CNN could not handle the order relationship in the amino-acid sequence, captured in the structural data. Öztürk et al. (2018), suggests avoiding this limitation by using an architecture more suitable for learning from long sequences of proteins, such as Long-Short Term Memory (LSTM).

#### WideDTA

To overcome the difficulty of modeling proteins using their sequences, the authors of DeepDTA attempted to improve the performance of DTBA prediction by developing a new method names WideDTA (made available through the e-print archives, arXiv) (Öztürk et al., 2019). WideDTA uses input data such as Ligand SMILES (LS) and amino acid sequences for protein sequences (PS), along with two other text-based information sources Ligand Maximum Common Substructure (LMCS) for drugs and Protein Domains and Motifs (PDM) based on PROSITE. Unlike DeepDTA, WideDTA represents PS and LS as a set of words instead of their full-length sequences. A word in PS is three-residues in the sequence, and a word in LS is 8-residues in the sequence. They claim, shorter lengths of residues that represent the features of the protein, are not detected using the full-length sequences due to the low signal to noise ratio. Thus, they proposed the word-based model instead of a character-based model. WideDTA is a CNN DL model that uses as input all four text-based information sources (PS, LS, LMCS, PDM) and predict binding affinity. It first uses the Keras Embedding layer (Erickson et al., 2017) to represent words with 128-dimensional dense vectors to fed integer encode inputs. Then, it sequentially applies two 1D-CNN layers with 32 and 64 filters, followed by a max-pooling layer by the activation function layer, ReLU:

$$\begin{array}{l} Features<-\;ReLU\left(pool\left(conv2\left(conv1\left(input\right)\right)\right)\right) \end{array}$$

Four models with the same architecture are used to extract features from each of the text-based information sources (PS, LS, LMCS, PDM). The output features from each model are then concatenated and fed to three fully connected (FC) layers (with two drop out layers to avoid overfitting problems) that predict the binding affinity.

#### PADME

PADME (Protein And Drug Molecule interaction prEdiction; Feng, 2019), is another DL-based method that applies drug-target features and fingerprints to different deep neural network architectures, to predict the binding affinity values. There are two versions of PADME. The first one called PADME-ECFP, uses the Extended-Connectivity Fingerprint (Rogers and Hahn, 2010) as input features that represent drugs. The second version called PADME-GraphConv integrates Molecular Graph Convolution (MGC) (Liu et al., 2019) into the model. This is done by adding one more Graph Convolution Neural Network (GCNN) layer (which is a generalization of CNN), which is used to learn the latent features of drugs from SMILES (i.e., from graphical representation). Both PADME versions use Protein Sequence Composition (PSC) (Michael Gromiha, 2011) descriptors, which contain rich information to represent the target proteins. After generating the feature vectors for each drug and target protein, a feature vector for each drug-target pair is fed into a simple FNN to predict the DTBA. Techniques used for regularization in the FNN includes dropout, early stopping, and batch normalization. The ReLU activation functions are used for the FC layers. The cross-validation (CV) process revealed the best hyperparameter (such as batch size, dropout rate, etc.) or combination thereof that is fixed and used to evaluate the test data.

#### DeepAffinity

DeepAffinity (Karimi et al., 2019) is a novel interpretable DL model for DTBA prediction, which relies only on using the SMILES representation of drugs and the structural property sequence (SPS) representation that annotates the sequence with structural information to represent the proteins. The SPS is better than other protein representations because it gives structural details and higher resolution of sequences (specifically among proteins in the same family), that benefits regression task. The SPS being better than other protein representations may also be as a consequence of the SPS sequence being shorter than other sequences. Both drug SMILES and protein SPS are encoded into embedding representations using a recurrent neural network (RNN) (Ghatak, 2019). RNN model named seq2seq (Shen and Huang, 2018) is used widely and successfully in natural language processing. The seq2seq model is an auto-encoder model that consists of a recurrent unit called “encoder” that maps sequence (i.e., SMILES/SPS) to a fixed dimensional vector, and other recurrent unit called “decoder” that map back the fixed-length vector into the original sequence (i.e., SMILES/SPS). These representation vectors that have been learned in an unsupervised fashion capture the non-linear mutual dependencies among compound atoms or protein residues. Subsequently, the RNN encoders and its attention mechanisms which are introduced to interpret the predictions, are coupled with a CNN model to develop feature vectors for the drugs and targets separately. The CNN model consists of a 1D convolution layer followed by a max-pooling layer. The output representation of the CNNs for both the drugs and targets are then concatenated and fed into FC layers to output the final results, DTBA values. The entire unified RNN-CNN pipeline, including data representation, embedding learning (unsupervised learning), and joint supervised learning trained from end to end, achieved very high accuracy results compared to ML models that use the same dataset (Karimi et al., 2019).

## Evaluation of the State-of-the-Art Methods

Since KronRLS, SimBoost, DeepDTA, DeepAffinity, WideDTA, and PADME are the only computational non-structure-based methods developed for prediction of DTBA to-date, we consider them the baseline methods. Here, we compare the performance of KronRLS, SimBoost, DeepDTA, WideDTA, and PADME, using the same benchmark datasets for evaluation. We excluded DeepAffinity from this comparison since it used different datasets which are based on BindingDB database (Liu et al., 2007). Also, when methods have more than one version, the comparison only includes the version that performs the best, based on identical evaluation metrics published for each method.

### Evaluation Metrics

The evaluation of the performance in these regression-based models uses five metrics:

- *Concordance Index (CI)*, first introduced by Gönen and Heller (2005), and was used first for evaluation in the development of KronRLS. *CI* is a ranking metric for continuous values that measure whether the predicted binding affinity values of two random drug-target pairs were predicted in the same order as their actual values were:
  (12) $$\begin{array}{l} CI\;=\frac{1}{Z}\underset{s_{i}>s_{j}}{\sum }h\left(b_{i}-b_{j}\right) \end{array}$$

where *b*_*i*_ is the prediction value for the larger affinity *s*_*i*_*, b*_*j*_ is the prediction value for the smaller affinity *s*_*j*_, *Z* is a normalization constant, and *h(x)* is the Heaviside step function (Davies, 2012), which is a discontinuous function defined as:

$$\begin{array}{l} h(x)\;=\{\begin{array}{l} \;1,\;x>0\\ 0.5,\;x=0\&\;\\ \;0,\;x<0 \end{array} \end{array}$$

where its value is either equal to zero when the input is negative or equal to one when the input is positive.

- *Mean Square Error (MSE)* (Wackerly et al., 2014) is commonly used as a loss function (i.e., error function) in regression task to measure how close the fitted line, that is represented by connecting the estimated values, is to the actual data points. The following formula defines the *MSE*, in which *P* denotes the prediction vector, *Y* denotes the vector of the actual outputs, and *n* is the number of samples. The square is used to ensure the negative values do not cancel the positive values. The value of *MSE* is close to zero, thus the smaller the *MSE*, the better the performance of the regressor (i.e., estimator):
  (13) $$\begin{array}{l} MSE=\overset{n}{\underset{i=1}{\sum }}\left(P_{i}-Y_{i}\right) \end{array}$$
- *Root Mean Squared Error (RMSE)* (Wackerly et al., 2014) is another metric to evaluate the regressor where it is the square root of *MSE*.
  (14) $$\begin{array}{l} RMSE=\sqrt[{2}]{MSE} \end{array}$$

*RMSE* is the distance, on average, of data points from the fitted line.

- *Pearson correlation coefficient (PCC)* (also known as Person's *R*; Kullback and Leibler, 1951) measures the difference between the actual values and the predicted values by measuring the linear correlation (association) between these two variables. The range of *PCC* is between +1 and −1, where +1 is a total positive linear correlation, −1 is a total negative linear correlation, and 0 is a non-linear correlation which indicates that there is no relationship between the actual values and the predicted values. The formula of *PCC* is defined as follows:
  (15) $$\begin{array}{l} PCC=\frac{cov\;\left(p,y\right)}{std\;\left(p\right)std\;\left(y\right)} \end{array}$$

where *cov* denotes the covariance between original values *y* and predicted values, and *std* denotes the standard deviation. The disadvantage is, *PCC* is only informative when used with variables that have linear correlation, as *PCC* results are misleading when used with non-linearly associated variables (Liu J. et al., 2016).

- *R-squared* (*R*^2^) (Kassambara, 2018) is the proportion of variation in the outcome that is explained by the predictor variables. The *R*^2^ corresponds to the squared correlation between the actual values and the predicted values in multiple regression models. The higher the *R*-squared, the better the model.

*CI* and *RMSE* are the only evaluation metrics reported by all the baseline methods; other metrics are reported but not by all the methods compared in this section. Also, *RMSE* and *MSE* represent the error function of the same type of error (i.e., mean square error) so reporting one of them is enough.

### Validation Settings

The performance of the methods in different prediction tasks is evaluated using various CV settings. The chosen setting can affect accuracy and make the evaluation results less realistic. KronRLS (Pahikkala et al., 2015) reported using three different CV settings that make the performance evaluation more accurate and realistic. One can split the input data (that is, how the set of drug-target pairs and their affinity labels, are split into training and testing dataset) in various ways, and this splitting of data defines the validation settings used. There are three main ways used to split input data:

- *Setting 1 (S1):* Random drug-target pair which correspond to regular *k*-fold CV that split the data into *k*-folds randomly, and keeps one of these folds for testing. That is, the training phase includes a significant portion of all the drug-target pairs, while the testing phase includes the remaining random pairs.
- *Setting 2 (S2):* New drug, which means the drug is missing from the training data corresponding to leave one drug out validation (LDO).
- *Setting 3 (S3):* New target, which means the target is missing from the training data corresponding to leave one target out validation (LTO).

KronRLS and PADME methods used these settings to evaluate subsequently developed DTI and DTBA prediction methods.

### Method Comparison

Tables 2, 3 summarize the performance of the baseline methods using all CV settings based on *RMSE* and *CI*, respectively. SimBoost and PADME reported *RMSE* in their respective publications. However, DeepDTA and WideDTA reported only *MSE*, so we calculated *RMSE* by taking the square root of their reported *MSE* values as defined by Equation (13). The KronRLS method did not report *RMSE* or *MSE*. However, the SimBoost paper calculated and reported *RMSE* for the KronRLS method (included in Table 2). Some of these baseline methods were only evaluated based on select datasets, while others only applied specific settings. All three dataset (Davis, Metz, and KIBA) were used to evaluate the performances of the SimBoost and PADME methods (based on self-reported results). The performance of PADME was also assessed using the ToxCast dataset. PADME is the first to use the ToxCast dataset. Moreover, PADME performances are reported using each dataset with the three settings (S1, S2, and S3) described above. However, SimBoost only provides its performance using one setting (S1) for each dataset.

**Table 2:** RMSE calculated using multiple settings for all baseline methods.

**Dataset**	**Setting**	**Method/reference of results**					
		**KronRLS**	**SimBoost**	**DeepDTA**	**PADME-EFP**	**PADME-GC**	**WideDTA**
Davis (*K_*d*_*)	*s1*	0.608^***^ 0.61562^**^ 0.57294^*^	**0.247** 0.53103^**^ 0.48197^*^	0.5109	**0.43219**	0.43225	0.5119
	*s2*	0.84048^*^	N/A	N/A	**0.78535**	0.80644	N/A
	*s3*	0.65964^*^	N/A	N/A	**0.56005**	0.57840	N/A
Metz (*K_*i*_*)	*s1*	0.562^***^0.78128^*^	0.1660 0.58154^*^	N/A	**0.55293**	0.59926	N/A
	*s2*	0.78429^*^	N/A	N/A	**0.71170**	0.74292	N/A
	*s3*	0.89889^*^	N/A	N/A	**0.79154**	0.81893	N/A
KIBA	*s1*	0.620^***^ 0.64109^**^ 0.65664^*^	**0.204** 0.47117^**^ 0.46888^*^	0.4405	0.43214	**0.418691**	0.42308
	*s2*	0.70243^*^	N/A	N/A	**0.60201**	0.62029	N/A
	*s3*	0.68111^*^	N/A	N/A	**0.61677**	0.62345	N/A
ToxCast	*s1*	N/A	N/A	N/A	**0.40563**	0.40779	N/A
	*s2*	N/A	N/A	N/A	0.4485	**0.44502**	N/A
	*s3*	N/A	N/A	N/A	**0.48698**	0.49439	N/A

The star symbols denote results that are not self-reported, i.e., the single star

* indicates that PADME reported the other methods results, double stars

** indicates that DeepDTA reported the other methods results, and the triple stars

*** *indicates that SimBoost reported the other methods results. Missing data are indicated with N/A. The best values for each setting are indicated in bold font*.

**Table 3:** CI across multiple datasets of all baseline methods.

**Dataset**	**Setting**	**Method/reference of results**					
		**KronRLS**	**SimBoost**	**DeepDTA**	**PADME-ECFP**	**PADME-GC**	**WideDTA**
Davis (*K_*d*_*)	*s1*	0.8830 0.8710^**^ 0.87578^*^	0.8840 0.872^**^ 0.8871^*^	0.8780	0.90388	**0.90389**	0.8860
	*s2*	**0.7480** 0.69245^*^	N/A	N/A	0.71630	0.72001	N/A
	*s3*	**0.8610** 0.80751^*^	N/A	N/A	0.85503	0.84483	N/A
Metz (*K_*i*_*)	*s1*	0.7930 0.748522^*^	**0.8510** 0.79439^*^	N/A	0.80756	0.79400	N/A
	*s2*	0.7360 0.70916^*^	N/A	N/A	**0.74240**	0.74104	N/A
	*s3*	0.6660 0.647^*^	N/A	N/A	0.69830	**0.70796**	N/A
KIBA	*s1*	0.782^**^ 0.7831^*^	0.8470 0.836^**^ 0.84046^*^	0.8630	0.85745	0.86370	**0.8750**
	*s2*	0.6890^*^	N/A	N/A	**0.77310**	0.75450	N/A
	*s3*	0.7122^*^	N/A	N/A	**0.77167**	0.76790	N/A
ToxCast	*s1*	N/A	N/A	N/A	0.79655	**0.79871**	N/A
	*s2*	N/A	N/A	N/A	0.72057	**0.7286**	N/A
	*s3*	N/A	N/A	N/A	0.68481	**0.69050**	N/A

The star symbols denote results that are not self-reported, i.e., the single star

* indicates that PADME reported the other methods results, and the double stars

** *indicates that DeepDTA reported the other methods results. Missing data are indicated with N/A. The best values for each setting are indicated in bold font*.

Thus, we added performance results at specific settings not found in the original manuscripts, as calculated and reported in studies published later, to compare differences in performance (these are denoted by stars ^*^, see Tables 2, 3 legend). In some instances, the results reported by other methods differ from the self-reported results. There are two reasons the results difference. The first is using different statistics of the datasets. For example, some methods, such as PADME, filter the KIBA dataset as well as adjusts the thresholds of other settings. The authors of PADME explained in their study, “*Because of the limitations of SimBoost and KronRLS, we filtered the datasets… Considering the huge compound similarity matrix required and the time-consuming matrix factorization used in SimBoost, it would be infeasible to work directly on the original KIBA dataset. Thus, we had to filter it rather aggressively so that the size becomes more manageable*.” Therefore, the authors of PADME reported different values for the *RMSE* scores of KronRLS and SimBoost, as shown in Table 2. The second reason is related to the CV settings such as the number of folds, the random seeds to split the data into training and testing, and the number of repeated experiments. The best values for each setting are indicated in bold font in Table 2.

Tables 2, 3 show that the SimBoost, DeepDTA, and WideDTA methods cannot handle the new drug and target settings (indicated by the missing data). From the methods that provide performances for all settings, we observe better performances using S1 setting (random pairs) compared to both S2 and S3 settings. The better performances acquired using S1 setting is expected for all methods and all datasets since it is the most informative. Better performances were also observed for S3 setting as compared to S2 setting, suggesting that the prediction of DTBA for new targets is more straightforward than the prediction of DTBA for new drugs (Pahikkala et al., 2015). However, we observe better performances for S2 setting than S3 setting when the number of targets is much lower than the number of drugs, as is the case for the Metz and ToxCast datasets.

From Tables 2, 3, we further conclude that overall, the DL-based methods outperform AI/ML-based methods in predicting DTBA. However, SimBoost error rate is smaller than other methods for specific datasets indicating that there are some characteristics of SimBoost and KronRLS that can improve prediction performance. In Table 4, we provide a comparison of all methods to summarize the characteristics of the methods shedding light on the differences that may be contributing to improved performance. The two AI/ML methods are similarity-based (SimBoost combines similarity and features), while the DL methods are features-based. These features were obtained automatically from the raw data using DL without doing any handcrafted feature engineering as in ML. Thus, developing DL-based methods for DTBA prediction eliminates the limitation of the ML methods associated with manual alteration of data. Different representations for both drugs and targets also present advantages discussed separately with each method above, and we provide recommendations concerning the use of different representation in the last section below.

**Table 4:** Baseline methods features.

**Characteristics**	**Methods**				
	**1. KronRLS**	**2. SimBoost**	**3. DeepDTA**	**4. WideDTA**	**5. PADME**
Datasets	Davis, Metz	Davis, Metz, Kiba	Davis, Kiba	Davis, Kiba	Davis, Metz, Kiba, ToxCast
ML/DL	AI/ML	AI/ML	DL	DL	DL
Similarity (OR) Feature based method	Similarity-based	Similarity and feature based	Feature-based	Feature-based	Feature-based
Drug representation (or features)	PubChem Sim Chemical kernels	PubChem Sim + statistical and network features	SMILES	SMILES + LMCS	SMILES / ECFP
Protein representation (or features)	SW sim score, Normalized SW sim score	SW sim score	aaseq	aaseq + PDM	PSC
NN type for features learning			CNN	two 1D-CNN	GCNN
NN type for prediction			3 FC layers	FC layer	Feedforward NN
Regressor/OR/activation function	KronRLS model	Gradient boosting model	ReLU	ReLU	ReLU
Validation setting	S1, S2, S3	S1	S1	S1	S1, S2, S3
Cross Validation	Repeated 10-folds CV, Nested CV, LDO-CV, LTO-CV	10 times 5 folds CV, LDO-CV, LTO-CV	5 folds CV	6 folds CV	5 folds CV, LDO-CV, LTO-CV
Performance metrics	CI, MSE	CI, RMSE	CI, MSE, PCC	CI, MSE, PCC	CI, RMSE, R^2^
Classification/Regression	Both	Both	Regression	Regression	Both
Year	2014	2017	2018	2019	2018

*ML, Machine Learning; DL, Deep Learning; Sim, Similarity; aaseq, amino-acid sequence; SPS, structural property sequence; PSC, protein sequence composition; PDM, protein domain and motif; ECFP, extended-connectivity fingerprint; LMCS, ligand maximum common substructure; KronRLS, Kronecker Regularized Least Square; CNN, convolutional neural network; GCNN, graph convolution neural network; RNN, recurrent neural network; FC, fully connected; ReLU, rectified linear unit; CV, cross validation; LDO, leave one drug out; LTO, leave one target out; MSE, Mean Square Error; RMSE, root square of mean square error; CI, concordance index; PCC, Pearson correlation coefficient*.

The comparison table also shows all DL-based methods reported up to now, used CNN to learn the features for both drugs and targets. The robust feature of CNN is its ability to capture local dependencies for both sequence and structure data. CNN is additionally computationally efficient since it uses unique convolution and pooling operations and performs parameter sharing (Defferrard et al., 2016). All DL methods use the same activation function, ReLU, which is the most widely used activation function for many reasons (Gupta, 2017). First, ReLU is non-linear function so it can easily backpropagate an error. Second, ReLU can have multiple layers of neurons, but it does not activate all these neurons at the same time. The last advantage of ReLU function is that it converts negative values of the input to zero values, and the neurons are not activated, so the network will be sparse which means easy and efficient of computation.

We can also observe from Table 4, that KronRLS, SimBoost, and PADME methods are suitable for both classification and regression problems. It is better to generalize the model to work on more than one application by making it suitable for both DTBA and DTIs predictions using the appropriate benchmark datasets and correct evaluation metrics.

## Limitations of AI/ML/DL-Based Methods

AI/ML/DL-based computational models developed for DTBA prediction show promising results. However, all such models suffer from limitations that if avoided, may improve performance.

### AI/ML-Based Methods

Similarity-based approaches used by these methods usually do not take into considerations the heterogeneous information defined in the relationship network. Avoiding this limitation requires integrating a feature-based approaches with the similarity-based approaches. Another limitation is that AI/ML-based models require extensive training, and each application requires specific training for the application-specific purpose. Moreover, shallow network-based methods with sequence data usually do not learn well some of the crucial features (such as distance correlation) that may be needed for accurate prediction.

### DL-Based Methods

The use of these methods is currently trending despite DL models creating “black boxes” that are difficult to interpret due to the learning features integrated into the data for modeling. Limitations faced with the use of DL models involve the requirement of the large amount of high-quality data, which are frequently kept private and is very expensive to generate. Not using a sufficiently large volume of high-quality data affects the reliability and performance of DL models. The other limitation is that the engineered features (generated automatically), are not intuitive, and the DL-based models developed lack rational interpretation of the biological/chemical aspects of the problem in question.

## Discussion

Here we attempt to extract useful insights from the characteristics of the methods developed for DTBA prediction, suggest possible future avenues to improve predictions, and highlight the existing problems that need a solution. Our recommendations are grouped under several sub-sections to focus on different aspects of improvements of prediction performance of DTBA.

### Using More Comprehensive Information

Integrating information from different sources of drug and target data can improve the prediction performance. These sources can include but are not limited to drug side-effects, drug-disease association, and drug interactions. For targets, examples of other sources of information are protein-protein interaction, protein-diseases association, and genotype-phenotype association. To the best of our knowledge, no method uses such information for DTBA prediction except KronRLS, which integrates some other sources of information in the form of similarity matrices. However, there are different DTIs prediction works that integrate different sources of information, which help in boosting the prediction performance. For example, some studies predicted DTIs by integrating drug side-effects information (Campillos et al., 2008; Mizutani et al., 2012), or drug-diseases interaction (Wang W. et al., 2014; Luo et al., 2017). Other studies used public gene expression data (Sirota et al., 2011), gene ontology (Tao et al., 2015), transcriptional response data (Iorio et al., 2010), or have integrated several of these resources (Alshahrani and Hoehndorf, 2018). DTBA prediction methods can benefit from these previous studies through integration of these different sources of information.

### Input Data Representation

Different representations can be used for both drugs and targets (see Table 4). For example, SMILES, max common substructure, and different kinds of fingerprints can be used to represent drugs. These representations significantly affect the prediction performance. Thus, it is essential to start with appropriate representations by deciding which features from these representations are intended to obtain. Each representation has its own advantages as discussed above when comparing methods.

### Similarity Calculation, Selection, and Information Fusion

There are several types of similarities that can be calculated using different sources of information, such as the multiple drug-drug similarities based on the chemical structures or based on side-effects. There are also other drug-drug similarities based on specific SMILES embeddings. The same goes for the target-target similarities, which can use other sources of information such as amino-acid sequence, nucleotide sequences, or protein-protein interaction network. Choosing suitable drug-drug and target-target similarities also contribute significantly to the prediction performance under different settings (either for DTBA or DTI prediction). If all similarities are combined, it will lead to introducing some noise as well as the most informative similarities will be affected by the less informative similarities. Thus, it is essential to apply a similarity selection method in order to select the most informative and robust subset of similarities among all similarities as introduced in Olayan et al. (2018). Integrating multiple similarities (i.e., a subset of similarities) has the advantage of complementary information for different similarities as well as avoiding dealing with a different scale. One could use the Similarity Network Fusion (SNF) (Wang B. et al., 2014) algorithm for data integration in a non-linear fashion to predict DTBA with multiple similarities. There are other integration algorithms or functions such as SUM, AVG, and MAX functions. Also, multi-view graph autoencoder algorithm (GAE) (Baskaran and Panchavarnam, 2019) proved its efficiency in integrating drug similarities (Ma T. et al., 2018).

### Integration of Computational Methods

Future *in silico* methods for DTBA prediction will benefit from the integration of diverse methods and approaches. Methods can be developed using different techniques, such as network analysis (Zong et al., 2019), matrix factorization (Ezzat et al., 2017), graph embeddings (Crichton et al., 2018), and more. Feature-based models and similarity-based models can be combined as well, as has been done in the SimBoost method. Furthermore, AI/ML/DL methods can be combined in different ways, (1) by combining some essential hand-crafted features from AI/ML and auto-generated features from DL, (2) using AI/ML for feature engineering and DL for prediction.

### Network Analysis and Graph Mining Techniques

Since graph mining and graph embedding approaches are very successful in the prediction of DTIs (Luo et al., 2017; Olayan et al., 2018), we can apply some of these techniques to DTBA. To apply this technique to DTBA we can formulate a weighted undirected heterogeneous graph G(V, E), where V is the set of vertices (i.e., drugs and targets), and E is the set of edges that represent the binding strength values. Multiple target-target similarities and drug-drug similarities can be integrated into the DTBA graph to construct a complete interaction network. After that, graph mining techniques such as Daspfind (Ba-Alawi et al., 2016) that calculate simple path scores between drug and target can be applied. Also, graph embedding techniques such as DeepWalk (Perozzi et al., 2014), node2vec (Grover and Leskovec, 2016), metapath2vec (Dong et al., 2017; Zhu et al., 2018), or Line (Tang et al., 2015) can be applied to the DTBA graph to obtain useful features for prediction. There are different graph embedding techniques that can be used for features learning and representation as summarized by Cai et al. (2018) and Goyal and Ferrara (2018a,b). To the best of our knowledge no published DTBA prediction method formulate the problem as a weighted graph and apply such techniques.

### Deep Learning

For the computational prediction of DTIs and DTBA, DL and features learning (i.e., embedding) are currently the most popular techniques since they are efficient in generating features and addressing scalability for large-scale data. DL techniques are capable of learning features of the drugs, targets, and the interaction network. Furthermore, when using heterogeneous information sources for drugs and targets, DL techniques can be applied to obtain additional useful features. DL techniques including different types of NN can extract useful features not just from the sequence-based representation of drug (i.e., SMILES) and protein (i.e., amino acid) as done by Öztürk et al. (2018, 2019), but also from the graph-based representation. For example, CNN, or GCNN can be applied on SMILES (that are considered graphs) to capture the structural information of the molecules (i.e., drugs). It is highly recommended to attempt to apply DL and feature learning techniques on graph-based techniques as well as a heterogeneous graph that combine different information about drugs and targets to enhance the DTBA predictive model. Several steps should be applied to develop a robust DL model: starting with selecting the suitable data representation, deciding about NN type and DL structures, then choosing the optimal hyperparameter set. The decisive advantage of the DL techniques worth mentioning is to implement the running of code on the Graphics Processing Unit (GPU). In terms of time complexity, DL-based methods that run on GPUs, drastically decrease computational time compared to running the method on a CPU. Guidelines to accelerate drug discovery applications using GPU as well as a comparison of recent GPU and CPU implementations are provided in Gawehn et al. (2018).

### Multi-Output Regression Methods

Given that DTBA can be measured using several output properties (e.g., IC_50_ and K_i_,), it is a laborious task to develop one model to predict each property individually. Therefore, it is much more efficient to generate a model that can predict several output properties, such as multi-output regression models (also known as multi-target regression), which aims at predicting several continuous values (Borchani et al., 2015). Multi-output regression differs from multi-label classification, which aims at predicting several binary labels (e.g., positive or negative; Gibaja and Ventura, 2014). Multi-output regression methods take into consideration correlations between output properties in addition to input conditions (e.g. organism and cell line). Borchani et al. (2015) recently wrote a review that covers more in-depth details regarding the multi-output regression methods. Moreover, Mei and Zhang (2019) demonstrated how multi-label classification methods could be applied for DTI prediction. In this study, each drug is considered a class label, and target genes are considered input data for training. To the best of our knowledge, multi-output regression methods have not been applied for DTBA prediction. The main challenge in applying multi-output regression to DTBA is missing data. Output properties (and sometimes input conditions) may not be available for all drug-target pairs in the dataset. However, several multi-label classification methods have been applied for handling missing data in multi-output datasets (Wu et al., 2014; Xu et al., 2014; Yu et al., 2014; Jain et al., 2016).

### Validation Settings

Overall, the methods further show that three settings for the CV are used to evaluate the prediction model. However, there are still many studies that only use the typical CV setting of random pair for evaluation (S1 setting), which leads to overoptimistic prediction results. Thus, models should be evaluated using all three settings. Models can also be evaluated using (a rarely used) fourth setting wherein both the drug and target are new (Pahikkala et al., 2015; Cichonska et al., 2017), and it is even better to evaluate the model under this setting as well, to see how good it is in predicting DTI when both the drug and the target are new. Evaluating the model under the four settings will avoid over-optimistic results. The CV is essential for adjusting the hyperparameters for both AI/ML and DL models. It is also essential to handle the overfitting problem. Overfitting happens when a model learns many details, including noise from the training data and fits the training data very well but cannot fit the test data well (Domingos, 2012). Overfitting can be evaluated by assessing how good the model is fitted to training data using some strategies that were recommended in Scior et al. (2009) and Raies and Bajic (2016) using two statistical parameters: *S*, standard error of estimation (Cronin and Schultz, 2003), and *R*^2^, coefficient of multiple determination (Gramatica, 2013), which will not be discussed in detail in this review.

### Evaluation Metrics

The choice of the suitable measure to evaluate DTBA prediction model is very important. Since DTBA prediction is a regression model, the evaluation metrics commonly used is *CI* and *RMSE*, as explained above. Nonetheless, other metrics (such as *R* and *PCC*) are partially used in assessment of DTBA prediction models. Using several metrics is essential as every metric carries disadvantages, which forces researchers to consider multiple evaluation metrics (Bajić, 2000) in performance evaluation to assess the model effectiveness in an accurate manner and from different perspectives. For example, *MSE* and *RMSE* are more sensitive to outliers (Chai and Draxler, 2014). *RMSE* is not a good indicator of average model performance and is a misleading indicator of average error. Thus, Mean Absolute Error (MAE) would be a better metric, as suggested by Willmott et al. (2009). So, it is better to have multiple evaluation metrics to get benefit from each one's strengths and evaluate the model from a different perspective.

## Conclusion

Both DTIs and DTBA predictions play a crucial role in the early stages of drug development and drug repurposing. However, it is more meaningful and informative to predict DTBA rather than predicting just on/off interaction between drug and target. An overview of the computational methods developed for DTBA prediction are summarized, but we specifically focused with more details on the recent AI/ML/DL-based methods developed to predict DTBA without the limitations imposed by 3D structural data. The available datasets for DTBA are summarized, and the benchmark datasets are discussed with details including definitions, sources, and statistics. For future research, computational prediction of DTBA remains an open problem. There is a lot of space to improve the existing computational methods from different angles as discussed in the recommendations. As the data is growing so fast, it is important to keep updating the prediction and updating evaluation datasets as well. After updating the data, it is necessary to customize, refine, and scale the current DTBA models, and to develop more efficient models as well.

## Author Contributions

MT designed the study and wrote the first draft of the manuscript. MT and AR designed the figures. AR and SA contributed to discussions and writing of specific sections of the manuscript. ME and VB supervised and critically revised the manuscript. All authors read and approved the final manuscript.

### Conflict of Interest

The authors declare that the research was conducted in the absence of any commercial or financial relationships that could be construed as a potential conflict of interest.

## Abbreviations

AI: artificial intelligence
ML: machine learning
DL: deep learning
Sim: similarity
aaseq: amino-acid sequence
SPS: structural property sequence
PSC: protein sequence composition
PDM: protein domain and motif
ECFP: extended-connectivity fingerprint
LMCS: ligand maximum common substructure
KronRLS: Kronecker regularized least square
CNN: convolutional neural network
GCNN: graph convolution neural network
FNN: feedforward neural network
ANN: artificial neural network
RNN: recurrent neural network
RBNN: radial basis function neural network
MNN: modular neural network
MLP: multilayer perceptron
RNN: recurrent neural network
FC: fully connected
ReLU: rectified linear unit
CV: cross validation
LDO: leave one drug out
LTO: leave one target out
MSE: mean square error
RMSE: root square of mean square error
CI: concordance index
PCC: Pearson correlation coefficient
NR: nuclear receptors
GPCR: G protein-coupled receptors
IC: ion channels
E: enzymes
KIBA: kinase inhibitor bioactivity.

## References

[B1] Abel R. Manas E. S. Friesner R. A. Farid R. S. Wang L. (2018). Modeling the value of predictive affinity scoring in preclinical drug discovery. Curr. Opin. Struct. Biol. 52, 103–110. 10.1016/j.sbi.2018.09.002 30321805

[B2] Agrawal P. Raghav P. K. Bhalla S. Sharma N. Raghava G. P. S. (2018). Overview of free software developed for designing drugs based on protein-small molecules interaction. Curr. Top. Med. Chem. 18, 1146–1167. 10.2174/1568026618666180816155131 30117394

[B3] Ahmed A. Smith R. D. Clark J. J. Dunbar J. B. Carlson H. A. (2015). Recent improvements to Binding MOAD: a resource for protein–ligand binding affinities and structures. Nucleic Acids Res. 43, D465–D469. 10.1093/nar/gku1088 25378330 PMC4383918

[B4] Ain Q. U. Aleksandrova A. Roessler F. D. Ballester P. J. (2015). Machine-learning scoring functions to improve structure-based binding affinity prediction and virtual screening. Wiley Interdiscip. Rev. Comput. Mol. Sci. 5, 405–424. 10.1002/wcms.1225 27110292 PMC4832270

[B5] Alshahrani M. Hoehndorf R. (2018). Drug repurposing through joint learning on knowledge graphs and literature. bioRXiv [Preprint]. 10.1101/385617

[B6] Andricopulo A. D. Ferreira L. L. G. (2019). Chemoinformatics approaches to structure- and ligand-based drug design. Front. Media SA. 9:1416. 10.3389/978-2-88945-744-1 30564124 PMC6289165

[B7] Antunes D. A. Abella J. R. Devaurs D. Rigo M. M. Kavraki L. E. (2019). Structure-based methods for binding mode and binding affinity prediction for peptide-MHC complexes. Curr. Top. Med. Chem. 18, 2239–2255. 10.2174/1568026619666181224101744 30582480 PMC6361695

[B8] Arrowsmith J. (2011). Trial watch: phase II failures: 2008–2010. Nat. Rev. Drug Discov. 10, 328–329. 10.1038/nrd3439 21532551

[B9] Ashtawy H. M. Mahapatra N. R. (2018). Task-specific scoring functions for predicting ligand binding poses and affinity and for screening enrichment. J. Chem. Inf. Model 58, 119–133. 10.1021/acs.jcim.7b00309 29190087

[B10] Ba-Alawi W. Soufan O. Essack M. Kalnis P. Bajic V. B. (2016). DASPfind: new efficient method to predict drug-target interactions. J. Cheminform. 8:15. 10.1186/s13321-016-0128-4 26985240 PMC4793623

[B11] Bachmann K. A. Lewis J. D. (2005). Predicting inhibitory drug—drug interactions and evaluating drug interaction reports using inhibition constants. Ann. Pharmacother. 39, 1064–1072. 10.1345/aph.1E508 15886285

[B12] Bajić V. B. (2000). Comparing the success of different prediction software in sequence analysis: a review. Brief. Bioinformatics 1, 214–228. 10.1093/bib/1.3.214 11465033

[B13] Baskaran S. Panchavarnam P. (2019). Data integration using through attentive multi-view graph auto-encoders. Int. J. Sci. Res. Comp. Sci. Eng. Inf. Technol. 5, 344–349. 10.32628/CSEIT195394

[B14] Benson M. L. Smith R. D. Khazanov N. A. Dimcheff B. Beaver J. Dresslar P.. (2007). Binding MOAD, a high-quality protein ligand database. Nucl. Acids Res. 36, D674–D678. 10.1093/nar/gkm911 18055497 PMC2238910

[B15] Berman H. M. Westbrook J. Feng Z. Gilliland G. Bhat T. N. Weissig H. (2000). The protein data bank. Nucleic Acids Res. 28, 235–242. 10.1093/nar/28.1.235 10592235 PMC102472

[B16] Block P. Sotriffer C. A. Dramburg I. Klebe G. (2006). AffinDB: a freely accessible database of affinities for protein-ligand complexes from the PDB. Nucleic Acids Res. 34, D522–526. 10.1093/nar/gkj039 16381925 PMC1347402

[B17] Borchani H. Varando G. Bielza C. Larrañaga P. (2015). A survey on multi-output regression. Wiley Interdiscip. Rev. Data Min. Knowl. Discov. 5, 216–233. 10.1002/widm.1157

[B18] Bulusu K. C. Guha R. Mason D. J. Lewis R. P. Muratov E. Motamedi Y. K.. (2016). Modelling of compound combination effects and applications to efficacy and toxicity: state-of-the-art, challenges and perspectives. Drug Discov. Today 21, 225–238. 10.1016/j.drudis.2015.09.003 26360051

[B19] Burlingham B. T. Widlanski T. S. (2003). An intuitive look at the relationship of K_i_ and IC50: a more general use for the dixon plot. J. Chem. Educ. 80:214 10.1021/ed080p214

[B20] Cai H. Zheng V. W. Chang K. C. (2018). A comprehensive survey of graph embedding: problems, techniques, and applications. IEEE Trans. Knowl. Data Eng. 30, 1616–1637. 10.1109/TKDE.2018.2807452

[B21] Campillos M. Kuhn M. Gavin A.-C. Jensen L. J. Bork P. (2008). Drug target identification using side-effect similarity. Science 321, 263–266. 10.1126/science.1158140 18621671

[B22] Chai T. Draxler R. R. (2014). Root mean square error (RMSE) or mean absolute error (MAE)?–Arguments against avoiding RMSE in the literature. Geosci. Model Dev. 7, 1247–1250. 10.5194/gmd-7-1247-2014

[B23] Chen H. Engkvist O. Wang Y. Olivecrona M. Blaschke T. (2018). The rise of deep learning in drug discovery. Drug Discov. Today 23, 1241–1250. 10.1016/j.drudis.2018.01.039 29366762

[B24] Chen X. Liu M. Gilson M. (2001). BindingDB: a web-accessible molecular recognition database. Comb. Chem. High Throughput Screen. 4, 719–725. 10.2174/1386207013330670 11812264

[B25] Cheng A. C. Coleman R. G. Smyth K. T. Cao Q. Soulard P. Caffrey D. R.. (2007). Structure-based maximal affinity model predicts small-molecule druggability. Nat. Biotechnol. 25, 71–75. 10.1038/nbt1273 17211405

[B26] Chou T. C. Talalay P. (1984). Quantitative analysis of dose-effect relationships: the combined effects of multiple drugs or enzyme inhibitors. Adv. Enzyme Regul. 22, 27–55. 10.1016/0065-2571(84)90007-4 6382953

[B27] Cichonska A. Ravikumar B. Parri E. Timonen S. Pahikkala T. Airola A.. (2017). Computational-experimental approach to drug-target interaction mapping: a case study on kinase inhibitors. PLoS Comput. Biol. 13:e1005678. 10.1371/journal.pcbi.1005678 28787438 PMC5560747

[B28] Colwell L. J. (2018). Statistical and machine learning approaches to predicting protein–ligand interactions. Curr. Opin. Struct. Biol. 49, 123–128. 10.1016/j.sbi.2018.01.006 29452923

[B29] Crichton G. Guo Y. Pyysalo S. Korhonen A. (2018). Neural networks for link prediction in realistic biomedical graphs: a multi-dimensional evaluation of graph embedding-based approaches. BMC Bioinformatics 19:176. 10.1186/s12859-018-2163-9 29783926 PMC5963080

[B30] Cronin M. T. D. Schultz T. W. (2003). Pitfalls in QSAR. J. Mol. Struct. 622, 39–51. 10.1016/S0166-1280(02)00616-4

[B31] Davies B. (2012). Integral Transforms and Their Applications. New York, NY: Springer Science and Business Media.

[B32] Davis M. I. Hunt J. P. Herrgard S. Ciceri P. Wodicka L. M. Pallares G.. (2011). Comprehensive analysis of kinase inhibitor selectivity. Nat. Biotechnol. 29, 1046–1051. 10.1038/nbt.1990 22037378

[B33] Defferrard M. Bresson X. Vandergheynst P. (2016). Convolutional Neural Networks on Graphs with Fast Localized Spectral Filtering, in Advances in Neural Information Processing Systems Vol. 29, eds Lee D. D. Sugiyama M. Luxburg U. V. Guyon I. Garnett R. (Barcelona: Curran Associates, Inc.), 3844–3852.

[B34] Degliesposti G. Portioli C. Parenti M. D. Rastelli G. (2011). BEAR, a novel virtual screening methodology for drug discovery. J. Biomol. Screen. 16, 129–133. 10.1177/1087057110388276 21084717

[B35] Deng W. Breneman C. Embrechts M. J. (2004). Predicting protein-ligand binding affinities using novel geometrical descriptors and machine-learning methods. J Chem. Inf. Comput. Sci. 44, 699–703. 10.1002/chin.200426198 15032552

[B36] Dimasi J. A. Hansen R. W. Grabowski H. G. (2003). The price of innovation: new estimates of drug development costs. J. Health Econ. 22, 151–185. 10.1016/S0167-6296(02)00126-1 12606142

[B37] Ding H. Takigawa I. Mamitsuka H. Zhu S. (2014). Similarity-based machine learning methods for predicting drug–target interactions: a brief review. Brief. Bioinformatics 15, 734–747. 10.1093/bib/bbt056 23933754

[B38] Domingos P. M. (2012). A few useful things to know about machine learning. Commun. ACM 55, 78–87. 10.1145/2347736.2347755

[B39] Dong Y. Chawla N. V. Swami A. (2017). Metapath2Vec: scalable representation learning for heterogeneous networks, in Proceedings of the 23rd ACM SIGKDD International Conference on Knowledge Discovery and Data Mining (Halifax, NS: ACM). 10.1145/3097983.3098036

[B40] Du X. Li Y. Xia Y.-L. Ai S.-M. Liang J. Sang P.. (2016). Insights into protein–ligand interactions: mechanisms, models, and methods. Int. J. Mol. Sci. 17:144. 10.3390/ijms17020144 26821017 PMC4783878

[B41] Dunbar J. B. Jr. Smith R. D. Damm-Ganamet K. L. Ahmed A. Esposito E. X. Delproposto J.. (2013). CSAR data set release 2012: ligands, affinities, complexes, and docking decoys. J. Chem. Inf. Model 53, 1842–1852. 10.1021/ci4000486 23617227 PMC3753885

[B42] Ekins S. (2016). The next era: deep learning in pharmaceutical research. Pharm. Res. 33, 2594–2603. 10.1007/s11095-016-2029-7 27599991 PMC5042864

[B43] Ekins S. Puhl A. C. Zorn K. M. Lane T. R. Russo D. P. Klein J. J.. (2019). Exploiting machine learning for end-to-end drug discovery and development. Nat. Mater. 18, 435–441. 10.1038/s41563-019-0338-z 31000803 PMC6594828

[B44] Emig D. Ivliev A. Pustovalova O. Lancashire L. Bureeva S. Nikolsky Y.. (2013). Drug target prediction and repositioning using an integrated network-based approach. PLoS ONE 8:e60618. 10.1371/journal.pone.0060618 23593264 PMC3617101

[B45] Erickson B. J. Korfiatis P. Akkus Z. Kline T. Philbrick K. (2017). Toolkits and libraries for deep learning. J. Digit. Imaging 30, 400–405. 10.1007/s10278-017-9965-6 28315069 PMC5537091

[B46] Ezzat A. Wu M. Li X. Kwoh C.-K. (2019). Computational prediction of drug-target interactions via ensemble learning. Methods Mol Biol. 1903, 239–254. 10.1007/978-1-4939-8955-3_14 30547446

[B47] Ezzat A. Wu M. Li X.-L. Kwoh C.-K. (2016). Drug-target interaction prediction via class imbalance-aware ensemble learning. BMC Bioinformatics 17:509. 10.1186/s12859-016-1377-y 28155697 PMC5259867

[B48] Ezzat A. Wu M. Li X.-L. Kwoh C.-K. (2018). Computational prediction of drug-target interactions using chemogenomic approaches: an empirical survey. Brief Bioinform. 20, 1337–1357. 10.1093/bib/bby002 29377981

[B49] Ezzat A. Zhao P. Wu M. Li X.-L. Kwoh C.-K. (2017). Drug-target interaction prediction with graph regularized matrix factorization. IEEE/ACM Trans. Comput. Biol. Bioinform. 14, 646–656. 10.1109/TCBB.2016.2530062 26890921

[B50] Feng Q. (2019). PADME: A Deep Learning-based Framework for Drug-Target Interaction Prediction (Master thesis), Simon Fraser University, Burnaby, BC, Canada.

[B51] Ferrero E. Dunham I. Sanseau P. (2017). *In silico* prediction of novel therapeutic targets using gene–disease association data. J. Transl. Med. 15:182. 10.1186/s12967-017-1285-6 28851378 PMC5576250

[B52] Ganotra G. K. Wade R. C. (2018). Prediction of drug–target binding kinetics by comparative binding energy analysis. ACS Med. Chem. Lett. 9, 1134–1139. 10.1021/acsmedchemlett.8b00397 30429958 PMC6231175

[B53] Gawehn E. Hiss J. A. Brown J. B. Schneider G. (2018). Advancing drug discovery via GPU-based deep learning. Expert Opin. Drug Discov. 13, 579–582. 10.1080/17460441.2018.1465407 29668343

[B54] Ghatak A. (2019). Recurrent neural networks (RNN) or sequence models. Deep Learn. R 1, 207–237. 10.1007/978-981-13-5850-0_8

[B55] Gibaja E. Ventura S. (2014). Multilabel learning: a review of the state of the art and ongoing research. Wiley Interdiscip. Rev. Data Min. Knowl. Discov. 4, 411–444. 10.1002/widm.1139

[B56] Gilson M. K. Liu T. Baitaluk M. Nicola G. Hwang L. Chong J. (2016). BindingDB in 2015: a public database for medicinal chemistry, computational chemistry and systems pharmacology. Nucleic Acids Res. 44, D1045–D1053. 10.1093/nar/gkv1072 26481362 PMC4702793

[B57] Gönen M. Heller G. (2005). Concordance probability and discriminatory power in proportional hazards regression. Biometrika 92, 965–970. 10.1093/biomet/92.4.965

[B58] Goyal P. Ferrara E. (2018a). GEM: a Python package for graph embedding methods. J. Open Source Softw. 3:876 10.21105/joss.00876

[B59] Goyal P. Ferrara E. (2018b). Graph embedding techniques, applications, and performance: a survey. Knowl. Based Syst. 151, 78–94. 10.1016/j.knosys.2018.03.022

[B60] Gramatica P. (2013). On the development and validation of QSAR models. Methods Mol. Biol. 930, 499–526. 10.1007/978-1-62703-059-5_21 23086855

[B61] Grover A. Leskovec J. (2016). node2vec: scalable feature learning for networks. KDD 2016, 855–864. 10.1145/2939672.2939754 27853626 PMC5108654

[B62] Guedes I. A. Pereira F. S. S. Dardenne L. E. (2018). Empirical scoring functions for structure-based virtual screening: applications, critical aspects, and challenges. Front. Pharmacol. 9:1089. 10.3389/fphar.2018.01089 30319422 PMC6165880

[B63] Gupta D. (2017). “Fundamentals of Deep Learning–Activation Functions and When to Use Them? [Online]. Available online at: https://www.analyticsvidhya.com/blog/2017/10/fundamentals-deep-learning-activationfunctions-when-to-use-them [accessed August, 1 2019].

[B64] He T. Heidemeyer M. Ban F. Cherkasov A. Ester M. (2017). SimBoost: a read-across approach for predicting drug–target binding affinities using gradient boosting machines. J. Cheminform. 9:24. 10.1186/s13321-017-0209-z 29086119 PMC5395521

[B65] Heck G. S. Pintro V. O. Pereira R. R. De Ávila M. B. Levin N. M. B. De Azevedo W. F. (2017). Supervised machine learning methods applied to predict ligand- binding affinity. Curr. Med. Chem. 24, 2459–2470. 10.2174/0929867324666170623092503 28641555

[B66] Hu L. Benson M. L. Smith R. D. Lerner M. G. Carlson H. A. (2005). Binding MOAD (mother of all databases). Proteins 60, 333–340. 10.1002/prot.20512 15971202

[B67] Huang S.-Y. Zou X. (2006). An iterative knowledge-based scoring function to predict protein–ligand interactions: I. Derivation of interaction potentials. J. Comput. Chem. 27, 1866–1875. 10.1002/jcc.20504 16983673

[B68] Hulme E. C. Trevethick M. A. (2010). Ligand binding assays at equilibrium: validation and interpretation. Br. J. Pharmacol. 161, 1219–1237. 10.1111/j.1476-5381.2009.00604.x 20132208 PMC3000649

[B69] Hutter M. C. (2009). *In silico* prediction of drug properties. Curr. Med. Chem. 16, 189–202. 10.2174/092986709787002736 19149571

[B70] Hutter M. C. (2018). The current limits in virtual screening and property prediction. Future Med. Chem. 10, 1623–1635. 10.4155/fmc-2017-0303 29953247

[B71] Iorio F. Bosotti R. Scacheri E. Belcastro V. Mithbaokar P. Ferriero R.. (2010). Discovery of drug mode of action and drug repositioning from transcriptional responses. Proc. Natl. Acad. Sci. U.S.A. 107, 14621–14626. 10.1073/pnas.1000138107 20679242 PMC2930479

[B72] Jain A. (2017). Deep Learning in Chemoinformatics Using Tensor Flow. (Doctoral dissertation). UC Irvine.

[B73] Jain H. Prabhu Y. Varma M. (2016). Extreme multi-label loss functions for recommendation, tagging, ranking & other missing label applications, in Proceedings of the 22nd ACM SIGKDD International Conference on Knowledge Discovery and Data Mining, 935–944. 10.1145/2939672.2939756

[B74] Jiang J. Wang N. Chen P. Zhang J. Wang B. (2017). DrugECs: an ensemble system with feature subspaces for accurate drug-target interaction prediction. Biomed Res. Int. 2017, 1–10. 10.1155/2017/6340316 28744468 PMC5514335

[B75] Jiménez J. Škalič M. Martínez-Rosell G. De Fabritiis G. (2018). KDEEP: protein–ligand absolute binding affinity prediction via 3D-convolutional neural networks. J. Chem. Inf. Model. 58, 287–296. 10.1021/acs.jcim.7b00650 29309725

[B76] Jing Y. Bian Y. Hu Z. Wang L. Xie X.-Q. S. (2018). Deep learning for drug design: an artificial intelligence paradigm for drug discovery in the big data era. AAPS J. 20:58 10.1208/s12248-018-0210-0 29603063 PMC6608578

[B77] Judson R. (2012). US EPA—ToxCast and the Tox21 program: perspectives. Toxicol. Lett. 211:S2 10.1016/j.toxlet.2012.03.016

[B78] Kalkatawi M. Magana-Mora A. Jankovic B. Bajic V. B. (2019). DeepGSR: an optimized deep-learning structure for the recognition of genomic signals and regions. Bioinformatics 35, 1125–1132. 10.1093/bioinformatics/bty752 30184052 PMC6449759

[B79] Karimi M. Wu D. Wang Z. Shen Y. (2019). DeepAffinity: interpretable deep learning of compound-protein affinity through unified recurrent and convolutional neural networks. Bioinformatics 35, 3329–3338. 10.1093/bioinformatics/btz111 30768156 PMC6748780

[B80] Kassambara A. (2018). Machine Learning Essentials: Practical Guide in R. STHDA.

[B81] Kontoyianni M. (2017). Docking and virtual screening in drug discovery. Methods Mol. Biol. 1647, 255–266. 10.1007/978-1-4939-7201-2_18 28809009

[B82] Krig S. (2016). Feature learning and deep learning architecture survey, in Computer Vision Metrics (Cham: Springer), 375–514. 10.1007/978-3-319-33762-3_10

[B83] Kullback S. Leibler R. A. (1951). On information and sufficiency. Ann. Math. Stat. 22, 79–86. 10.1214/aoms/1177729694

[B84] Kundu I. Paul G. Banerjee R. (2018). A machine learning approach towards the prediction of protein–ligand binding affinity based on fundamental molecular properties. RSC Adv. 8, 12127–12137. 10.1039/C8RA00003D PMC9079328 35539386

[B85] Kurgan L. Wang C. (2018). Survey of similarity-based prediction of drug-protein interactions. Curr. Med. Chem. 26:1 10.2174/0929867326666190808154841 31393241

[B86] Leach A. R. Shoichet B. K. Peishoff C. E. (2006). Prediction of Protein-Ligand Interactions. Docking and scoring: successes and gaps. J. Med. Chem. 49, 5851–5855. 10.1021/jm060999m 17004700

[B87] Lee A. Lee K. Kim D. (2016). Using reverse docking for target identification and its applications for drug discovery. Expert Opin. Drug Discov. 11, 707–715. 10.1080/17460441.2016.1190706 27186904

[B88] Li J. Fu A. Zhang L. (2019). An overview of scoring functions used for protein–ligand interactions in molecular docking. Interdiscip. Sci. 11, 320–328. 10.1007/s12539-019-00327-w 30877639

[B89] Li Q. Shah S. (2017). Structure-Based Virtual Screening. Methods Mol. Biol. 1558, 111–124. 10.1007/978-1-4939-6783-4_5 28150235

[B90] Li Y. Huang C. Ding L. Li Z. Pan Y. Gao X. (2019). Deep learning in bioinformatics: introduction, application, and perspective in the big data era. Methods 166, 4–21. 10.1101/563601 31022451

[B91] Lima A. N. Philot E. A. Trossini G. H. G. Scott L. P. B. Maltarollo V. G. Honorio K. M. (2016). Use of machine learning approaches for novel drug discovery. Expert. Opin. Drug Discov. 11, 225–239. 10.1517/17460441.2016.1146250 26814169

[B92] Liu J. Tang W. Chen G. Lu Y. Feng C. Tu X. M. (2016). Correlation and agreement: overview and clarification of competing concepts and measures. Shanghai Arch. Psychiatry 28, 115–120. 10.11919/j.issn.1002-0829.216045 27605869 PMC5004097

[B93] Liu K. Sun X. Jia L. Ma J. Xing H. Wu J.. (2019). Chemi-Net: a molecular graph convolutional network for accurate drug property prediction. Int. J. Mol. Sci. 20:3389. 10.3390/ijms20143389 31295892 PMC6678642

[B94] Liu T. Lin Y. Wen X. Jorissen R. N. Gilson M. K. (2007). BindingDB: a web-accessible database of experimentally determined protein-ligand binding affinities. Nucleic Acids Res. 35, D198–D201. 10.1093/nar/gkl999 17145705 PMC1751547

[B95] Liu W. Wang Z. Liu X. Zeng N. Liu Y. Alsaadi F. E. (2017). A survey of deep neural network architectures and their applications. Neurocomputing 234, 11–26. 10.1016/j.neucom.2016.12.038

[B96] Liu Y. Wu M. Miao C. Zhao P. Li X.-L. (2016). Neighborhood regularized logistic matrix factorization for drug-target interaction prediction. PLoS Comput. Biol. 12:e1004760. 10.1371/journal.pcbi.1004760 26872142 PMC4752318

[B97] Liu Y. Xu Z. Yang Z. Chen K. Zhu W. (2013). A knowledge-based halogen bonding scoring function for predicting protein-ligand interactions. J. Mol. Model. 19, 5015–5030. 10.1007/s00894-013-2005-7 24072554

[B98] Lu J. Lu D. Fu Z. Zheng M. Luo X. (2018). Machine learning-based modeling of drug toxicity. Methods Mol. Biol. 1754, 247–264. 10.1007/978-1-4939-7717-8_15 29536448

[B99] Luo Y. Zhao X. Zhou J. Yang J. Zhang Y. Kuang W.. (2017). A network integration approach for drug-target interaction prediction and computational drug repositioning from heterogeneous information. Nat. Commun. 8:573. 10.1038/s41467-017-00680-8 28924171 PMC5603535

[B100] Ma T. Xiao C. Zhou J. Wang F. (2018). Drug similarity integration through attentive multi-view graph auto-encoders, in Proceedings of the Twenty-Seventh International Joint Conference on Artificial Intelligence, 3477–3483.

[B101] Ma W. Yang L. He L. (2018). Overview of the detection methods for equilibrium dissociation constant KD of drug-receptor interaction. J. Pharm. Anal. 8, 147–152. 10.1016/j.jpha.2018.05.001 29922482 PMC6004624

[B102] Mei S. Zhang K. (2019). A multi-label learning framework for drug repurposing. Pharmaceutics 11:466. 10.3390/pharmaceutics11090466 31505805 PMC6781509

[B103] Metz J. T. Johnson E. F. Soni N. B. Merta P. J. Kifle L. Hajduk P. J. (2011). Navigating the kinome. Nat. Chem. Biol. 7, 200–202. 10.1038/nchembio.530 21336281

[B104] Michael Gromiha M. (2011). Protein Bioinformatics: From Sequence to Function. New Delhi: Academic Press.

[B105] Michelucci U. (2018). Feedforward neural networks. Appl. Deep Learn. 1, 83–136. 10.1007/978-1-4842-3790-8_3

[B106] Mizutani S. Pauwels E. Stoven V. Goto S. Yamanishi Y. (2012). Relating drug–protein interaction network with drug side effects. Bioinformatics 28, i522–i528. 10.1093/bioinformatics/bts383 22962476 PMC3436810

[B107] Mutowo P. Bento A. P. Dedman N. Gaulton A. Hersey A. Lomax J.. (2016). A drug target slim: using gene ontology and gene ontology annotations to navigate protein-ligand target space in ChEMBL. J. Biomed. Semantics 7:59. 10.1186/s13326-016-0102-0 27678076 PMC5039825

[B108] Nair V. Hinton G. E. (2010). Rectified linear units improve restricted boltzmann machines, in Proceedings of the 27th International Conference on Machine Learning (ICML-10) (Haifa). Available online at: https://web.cs.toronto.edu/

[B109] Newman M. (2018). Mathematics of networks, in Networks (Oxford: Oxford University Press). 10.1093/oso/9780198805090.003.0006

[B110] Olayan R. S. Ashoor H. Bajic V. B. (2018). DDR: efficient computational method to predict drug–target interactions using graph mining and machine learning approaches. Bioinformatics 34, 3779–3779. 10.1093/bioinformatics/bty417 29917050 PMC6198857

[B111] Öztürk H. Özgür A. Ozkirimli E. (2018). DeepDTA: deep drug-target binding affinity prediction. Bioinformatics 34, i821–i829. 10.1093/bioinformatics/bty593 30423097 PMC6129291

[B112] Öztürk H. Ozkirimli E. Özgür A. (2019). WideDTA: prediction of drug-target binding affinity. arXiv:1902.04166. Available online at: https://arxiv.org/abs/1902.04166 (accessed July 15, 2019).

[B113] Pahikkala T. Airola A. Pietilä S. Shakyawar S. Szwajda A. Tang J.. (2015). Toward more realistic drug-target interaction predictions. Brief. Bioinformatics. 16, 325–337. 10.1093/bib/bbu010 24723570 PMC4364066

[B114] Pahikkala T. Okser S. Airola A. Salakoski T. Aittokallio T. (2012a). Wrapper-based selection of genetic features in genome-wide association studies through fast matrix operations. Algorithms Mol. Biol. 7:11. 10.1186/1748-7188-7-11 22551170 PMC3606421

[B115] Pahikkala T. Suominen H. Boberg J. (2012b). Efficient cross-validation for kernelized least-squares regression with sparse basis expansions. Mach. Learn. 87, 381–407. 10.1007/s10994-012-5287-6

[B116] Perozzi B. Al-Rfou R. Skiena S. (2014). DeepWalk: online learning of social representations, in Proceedings of the 20th ACM SIGKDD International Conference on Knowledge Discovery and Data Mining (New York, NY: ACM). 10.1145/2623330.2623732

[B117] Puvanendrampillai D. Mitchell J. B. O. (2003). L/D protein ligand database (PLD): additional understanding of the nature and specificity of protein-ligand complexes. Bioinformatics 19, 1856–1857. 10.1093/bioinformatics/btg243 14512362

[B118] Raies A. B. Bajic V. B. (2016). *In silico* toxicology: computational methods for the prediction of chemical toxicity. Wiley Interdiscip. Rev. Comput. Mol. Sci. 6, 147–172. 10.1002/wcms.1240 27066112 PMC4785608

[B119] Raies A. B. Bajic V. B. (2018). *In silico* toxicology: comprehensive benchmarking of multi-label classification methods applied to chemical toxicity data. Wiley Interdiscip. Rev. Comput. Mol. Sci. 8:e1352. 10.1002/wcms.1352 29780432 PMC5947741

[B120] Raschka S. Scott A. M. Huertas M. Li W. Kuhn L. A. (2018). Automated inference of chemical discriminants of biological activity. Methods Mol. Biol. 1762, 307–338. 10.1007/978-1-4939-7756-7_16 29594779

[B121] Rayhan F. Ahmed S. Farid D. M. Dehzangi A. Shatabda S. (2019). CFSBoost: cumulative feature subspace boosting for drug-target interaction prediction. J. Theor. Biol. 464, 1–8. 10.1016/j.jtbi.2018.12.024 30578798

[B122] Rayhan F. Ahmed S. Shatabda S. Farid D. M. Mousavian Z. Dehzangi A.. (2017). iDTI-ESBoost: identification of drug target interaction using evolutionary and structural features with boosting. Sci. Rep. 7:17731. 10.1038/s41598-017-18025-2 29255285 PMC5735173

[B123] Roche O. Kiyama R. Brooks C. L. 3^rd^. (2001). Ligand-protein database: linking protein-ligand complex structures to binding data. J. Med. Chem. 44, 3592–3598. 10.1021/jm000467k 11606123

[B124] Rogers D. Hahn M. (2010). Extended-connectivity fingerprints. J. Chem. Inf. Model. 50, 742–754. 10.1021/ci100050t 20426451

[B125] Salahudeen M. S. Nishtala P. S. (2017). An overview of pharmacodynamic modelling, ligand-binding approach and its application in clinical practice. Saudi Pharm. J. 25, 165–175. 10.1016/j.jsps.2016.07.002 28344466 PMC5355565

[B126] Scarpino A. Ferenczy G. G. Keseru G. M. (2018). Comparative evaluation of covalent docking tools. J. Chem. Inf. Model. 58, 1441–1458. 10.1021/acs.jcim.8b00228 29890081

[B127] Schmidhuber J. (2015). Deep learning in neural networks: an overview. Neural Netw. 61, 85–117. 10.1016/j.neunet.2014.09.003 25462637

[B128] Scior T. Medina-Franco J. L. Do Q. T. Martínez-Mayorga K. Yunes Rojas J. A.. (2009). How to recognize and workaround pitfalls in QSAR studies: a critical review. Curr. Med. Chem. 16, 4297–4313. 10.2174/092986709789578213 19754417

[B129] Shen M. Huang R. (2018). A personal conversation assistant based on Seq2seq with Word2vec cognitive map, in 2018 7th International Congress on Advanced Applied Informatics (IIAI-AAI) (Yonago: IEEE CPS). 10.1109/IIAI-AAI.2018.00136

[B130] Sirota M. Dudley J. T. Kim J. Chiang A. P. Morgan A. A. Sweet-Cordero A.. (2011). Discovery and preclinical validation of drug indications using compendia of public gene expression data. Sci. Transl. Med. 3:96ra77. 10.1126/scitranslmed.3001318 21849665 PMC3502016

[B131] Sledz P. Caflisch A. (2018). Protein structure-based drug design: from docking to molecular dynamics. Curr. Opin. Struct. Biol. 48, 93–102. 10.1016/j.sbi.2017.10.010 29149726

[B132] Smith R. D. Clark J. J. Ahmed A. Orban Z. J. Dunbar J. B. Jr. Carlson H. A. (2019). Updates to binding MOAD (mother of all databases): polypharmacology tools and their utility in drug repurposing. J. Mol. Biol. 431, 2423–2433. 10.1016/j.jmb.2019.05.024 31125569 PMC6589129

[B133] Smith R. D. Dunbar J. B. Jr. Ung P. M.-U. Esposito E. X. Yang C.-Y. Wang S.. (2011). CSAR benchmark exercise of 2010: combined evaluation across all submitted scoring functions. J. Chem. Inf. Model. 51, 2115–2131. 10.1021/ci200269q 21809884 PMC3186041

[B134] Sotriffer C. Matter H. (2011). The challenge of affinity prediction: scoring functions for structure-based virtual screening. Methods Princ. Med. Chem. 1, 177–221. 10.1002/9783527633326.ch7

[B135] Srivastava N. Hinton G. Krizhevsky A. Sutskever I. Salakhutdinov R. (2014). Dropout: a simple way to prevent neural networks from overfitting. J. Mach. Learn. Res. 15, 1929–1958.

[B136] Stefan M. I. Le Novère N. (2013). Cooperative binding. PLoS Comput. Biol. 9:e1003106. 10.1371/journal.pcbi.1003106 23843752 PMC3699289

[B137] Sun H. Duan L. Chen F. Liu H. Wang Z. Pan P.. (2018). Assessing the performance of MM/PBSA and MM/GBSA methods. 7. Entropy effects on the performance of end-point binding free energy calculation approaches. Phys. Chem. Chem. Phys. 20, 14450–14460. 10.1039/C7CP07623A 29785435

[B138] Tang J. Qu M. Wang M. Zhang M. Yan J. Mei Q. (2015). LINE: large-scale information network embedding, in Proceedings of the 24th International Conference on World Wide Web (Florence: International World Wide Web Conferences Steering Committee). 10.1145/2736277.2741093

[B139] Tang J. Szwajda A. Shakyawar S. Xu T. Hintsanen P. Wennerberg K.. (2014). Making sense of large-scale kinase inhibitor bioactivity data sets: a comparative and integrative analysis. J. Chem. Inf. Model. 54, 735–743. 10.1021/ci400709d 24521231

[B140] Tang Z. Roberts C. C. Chia-En A. C. (2017). Understanding ligand-receptor non-covalent binding kinetics using molecular modeling. Front. Biosci. 22:960. 10.2741/4527 27814657 PMC5470370

[B141] Tao C. Sun J. Jim Zheng W. Chen J. Xu H. (2015). Colorectal cancer drug target prediction using ontology-based inference and network analysis. Database. 2015:bav015. 10.1093/database/bav015 25818893 PMC4375358

[B142] Tatar G. Taskin Tok T. (2019). Structure prediction of eukaryotic elongation factor-2 kinase and identification of the binding mechanisms of its inhibitors: homology modeling, molecular docking and molecular dynamics simulation. J. Biomol. Struct. Dyn. 18, 1–16. 10.1080/07391102.2019.1592024 30880628

[B143] Trosset J.-Y. Cavé C. (2019). *In silico* drug–target profiling, in Target Identification and Validation in Drug Discovery: Methods and Protocols. eds Moll J. Carotta S. (New York, NY: Springer, 89–103. 10.1007/978-1-4939-9145-7_6 30912017

[B144] Tsubaki M. Tomii K. Sese J. (2019). Compound-protein interaction prediction with end-to-end learning of neural networks for graphs and sequences. Bioinformatics 35, 309–318. 10.1093/bioinformatics/bty535 29982330

[B145] Vakil V. Trappe W. (2019). Drug combinations: mathematical modeling and networking methods. Pharmaceutics 11:e208. 10.3390/pharmaceutics11050208 31052580 PMC6571786

[B146] Vallone A. D'alessandro S. Brogi S. Brindisi M. Chemi G. Alfano G.. (2018). Antimalarial agents against both sexual and asexual parasites stages: structure-activity relationships and biological studies of the Malaria Box compound 1-[5-(4-bromo-2-chlorophenyl) furan-2-yl]-N-[(piperidin-4-yl) methyl] methanamine (MMV019918) and analogues. Eur. J. Med. Chem. 150, 698–718. 10.1016/j.ejmech.2018.03.024 29571157 PMC5902032

[B147] Vamathevan J. Clark D. Czodrowski P. Dunham I. Ferran E. Lee G.. (2019). Applications of machine learning in drug discovery and development. Nat. Rev. Drug Discov. 18, 463–477. 10.1038/s41573-019-0024-5 30976107 PMC6552674

[B148] Van Laarhoven T. Nabuurs S. B. Marchiori E. (2011). Gaussian interaction profile kernels for predicting drug–target interaction. Bioinformatics 27, 3036–3043. 10.1093/bioinformatics/btr500 21893517

[B149] Vert J. P. Jacob L. (2008). Machine learning for *in silico* virtual screening and chemical genomics: new strategies. Comb. Chem. High Throughput Screen 11, 677–685. 10.2174/138620708785739899 18795887 PMC2748698

[B150] Wackerly D. Mendenhall W. Scheaffer R. L. (2014). Mathematical Statistics With Applications. Belmont, NC: BROOKS/COLE Cengage Learning.

[B151] Wan F. Hong L. Xiao A. Jiang T. Zeng J. (2019). NeoDTI: neural integration of neighbor information from a heterogeneous network for discovering new drug–target interactions. Bioinformatics 35, 104–111. 10.1093/bioinformatics/bty543 30561548

[B152] Wang B. Mezlini A. M. Demir F. Fiume M. Tu Z. Brudno M.. (2014). Similarity network fusion for aggregating data types on a genomic scale. Nat. Methods 11, 333–337. 10.1038/nmeth.2810 24464287

[B153] Wang F. Yang W. Hu X. (2019). Discovery of high affinity receptors for dityrosine through inverse virtual screening and docking and molecular dynamics. Int. J. Mol. Sci. 20:115. 10.3390/ijms20010115 30597963 PMC6337580

[B154] Wang K. Sun J. Zhou S. Wan C. Qin S. Li C.. (2013). Prediction of drug-target interactions for drug repositioning only based on genomic expression similarity. PLoS Comput. Biol. 9:e1003315. 10.1371/annotation/958d4c23-4f1e-4579-b6ef-8ae1f828b1dd 24244130 PMC3820513

[B155] Wang R. Fang X. Lu Y. Wang S. (2004). The PDBbind database: collection of binding affinities for protein–ligand complexes with known three-dimensional structures. J. Med. Chem. 47, 2977–2980. 10.1021/jm030580l 15163179

[B156] Wang R. Fang X. Lu Y. Yang C.-Y. Wang S. (2005). The PDBbind database: methodologies and updates. J. Med. Chem. 48, 4111–4119. 10.1021/jm048957q 15943484

[B157] Wang W. Yang S. Zhang X. Li J. (2014). Drug repositioning by integrating target information through a heterogeneous network model. Bioinformatics 30, 2923–2930. 10.1093/bioinformatics/btu403 24974205 PMC4184255

[B158] Weiland G. A. Molinoff P. B. (1981). Quantitative analysis of drug-receptor interactions: I. Determination of kinetic and equilibrium properties. Life Sci. 29, 313–330. 10.1016/0024-3205(81)90324-6 6116136

[B159] Weininger D. (1988). SMILES, a chemical language and information system. 1. Introduction to methodology and encoding rules. J. Chem. Inf. Model. 28, 31–36. 10.1021/ci00057a005

[B160] Weininger D. (1990). SMILES. 3. DEPICT. Graphical depiction of chemical structures. J. Chem. Inf. Model. 30, 237–243. 10.1021/ci00067a005

[B161] Westbrook J. Feng Z. Chen L. Yang H. Berman H. M. (2003). The Protein Data Bank and structural genomics. Nucleic Acids Res. 31, 489–491. 10.1093/nar/gkg068 12520059 PMC165515

[B162] Willmott C. J. Matsuura K. Robeson S. M. (2009). Ambiguities inherent in sums-of-squares-based error statistics. Atmos. Environ. 43, 749–752. 10.1016/j.atmosenv.2008.10.005

[B163] Wu B. Liu Z. Wang S. Hu B.-G. Ji Q. (2014). Multi-label learning with missing labels, in 2014 22nd International Conference on Pattern Recognition, 1964–1968. 10.1109/ICPR.2014.343

[B164] Wu H. (2009). Global stability analysis of a general class of discontinuous neural networks with linear growth activation functions. Inf. Sci. 179, 3432–3441. 10.1016/j.ins.2009.06.006

[B165] Xu L. Wang Z. Shen Z. Wang Y. Chen E. (2014). Learning low-rank label correlations for multi-label classification with missing labels, in 2014 IEEE International Conference on Data Mining, 1067–1072. 10.1109/ICDM.2014.125

[B166] Yamanishi Y. Araki M. Gutteridge A. Honda W. Kanehisa M. (2008). Prediction of drug-target interaction networks from the integration of chemical and genomic spaces. Bioinformatics 24, i232–i240. 10.1093/bioinformatics/btn162 18586719 PMC2718640

[B167] Yu H.-F. Jain P. Kar P. Dhillon I. (2014). Large-scale multi-label learning with missing labels, in International Conference on Machine Learning, 593–601. 28650814

[B168] Zhu S. Bing J. Min X. Lin C. Zeng X. (2018). Prediction of drug-gene interaction by using Metapath2vec. Front. Genet. 9:248. 10.3389/fgene.2018.00248 30108606 PMC6079268

[B169] Zhu S. Okuno Y. Tsujimoto G. Mamitsuka H. (2005). A probabilistic model for mining implicit ‘chemical compound-gene' relations from literature. Bioinformatics 21, ii245–ii251. 10.1093/bioinformatics/bti1141 16204113

[B170] Zong N. Kim H. Ngo V. Harismendy O. (2017). Deep mining heterogeneous networks of biomedical linked data to predict novel drug-target associations. Bioinformatics 33, 2337–2344. 10.1093/bioinformatics/btx160 28430977 PMC5860112

[B171] Zong N. Wong R. S. N. Ngo V. (2019). Tripartite network-based repurposing method using deep learning to compute similarities for drug-target prediction. Methods Mol. Biol. 1903, 317–328. 10.1007/978-1-4939-8955-3_19 30547451

